# Targeting HIF-2α in glioblastoma reshapes the immune infiltrate and enhances response to immune checkpoint blockade

**DOI:** 10.1007/s00018-025-05642-8

**Published:** 2025-03-17

**Authors:** Felipe I. Espinoza, Stoyan Tankov, Sylvie Chliate, Joana Pereira Couto, Eliana Marinari, Thibaud Vermeil, Marc Lecoultre, Nadia El Harane, Valérie Dutoit, Denis Migliorini, Paul R. Walker

**Affiliations:** 1 Translational Research Centre in Oncohaematology, Faculty of Medicine, University of Geneva, Geneva, Switzerland; 2 Swiss Cancer Center Léman, Geneva, Lausanne, Switzerland; 3 Agora Cancer Research Center, Lausanne, Switzerland

**Keywords:** Glioma, Tumor microenvironment, Hypoxia inducible factor, HIF-2, Treg, Tumor-associated macrophage, Microglia, Immune checkpoint inhibitors, Brain tumors, Immunotherapy

## Abstract

Glioblastoma (GBM) is an aggressive primary brain tumor with dismal clinical prognosis and resistance to current therapies. GBM progression is facilitated by the tumor microenvironment (TME), with an immune infiltrate dominated by tumor-associated microglia/macrophages (TAMs) and regulatory T cells (Tregs). The TME is also characterized by hypoxia and the expression of hypoxia-inducible factors (HIFs), with HIF-2α emerging as a potential regulator of tumor progression. However, its role in GBM immunosuppression remains unknown. Here, we investigate HIF-2α and the use of the HIF-2α inhibitor PT2385 to modulate the TME in the immunocompetent GL261 mouse GBM model. PT2385 administration in vivo decreased tumor volume and prolonged survival of tumor-bearing mice, without affecting GL261 viability in vitro. Notably, HIF-2α inhibition alleviated the immunosuppressive TME and synergized with immune checkpoint blockade (ICB) using αPD-1 and αTIM-3 antibodies to promote long-term survival. Comprehensive analysis of the immune infiltrate through single-cell RNA sequencing and flow cytometry revealed that combining PT2385 with ICB reduced numbers of pro-tumoral macrophages and Tregs while increasing numbers of microglia, with a corresponding transcriptional modulation towards an anti-tumoral profile of these TAMs. In vitro, deletion of HIF-2α in microglia impeded their polarization towards a pro-tumoral M2-like profile, and its inhibition impaired Treg migration. Our results show that targeting HIF-2α can switch an immunosuppressive TME towards one that favors a robust and sustained response to ICB based immunotherapy. These findings establish that clinically relevant HIF-2α inhibitors should be explored not only in malignancies with defects in the HIF-2α axis, but also in those exhibiting an immunosuppressive TME that limits immunotherapy responsiveness.

**Supplementary Information:**

The online version contains supplementary material available at 10.1007/s00018-025-05642-8.

## Introduction

GBM is the most common and the most aggressive malignant primary brain tumor [1]. Despite the current standard of care, the median overall survival (OS) is ~ 15 months [2]. Given the poor functionality of GBM-infiltrating T cells, ICB therapies, particularly anti-PD-1/PD-L1 have been being explored in clinical trials [3–5]. However, their clinical benefits remain limited and variable [6, 7]. GBM responsiveness to ICB has been suggested to be linked to tumor mutational burden, defects in DNA replicative stress response, presence of certain rare mutations in the glioma cells and, importantly, tumor-immune crosstalk [8]. Additional immune checkpoints, such as TIM-3, LAG-3, and CTLA-4, have also been targeted, often in combination with anti-PD-1, showing effectiveness in some mouse glioma models [5, 9–13]. These findings highlight the complexity of immune responses and the critical influence of tumor heterogeneity on the efficacy of GBM immunotherapy.

The main limitation of ICB efficacy is that T-cell infiltration within the GBM TME is variable and generally limited [14, 15]. Furthermore, T cells within the TME undergo exhaustion, characterized by upregulation of immune checkpoint molecules such as PD-1, TIM-3, CTLA-4, and LAG-3, impairing anti-tumor functions [16]. In contrast, infiltration of Tregs has been shown to correlate with poor prognosis [17, 18]. Another major hurdle for ICB efficacy is the presence of a highly immunosuppressive TME, with a complex and dynamic ecosystem comprising cellular and non-cellular elements [4, 14, 19]. Among the cells contributing to GBM immunosuppression, TAMs comprising tissue-resident microglia, monocyte-derived macrophages and border-associated macrophages (BAMs) are the primary immune infiltrating cells within gliomas, constituting up to 50% of the tumor mass [20, 21]. Microglia and BAMs are the brain’s resident macrophages; however, blood-brain barrier (BBB) disruption during gliomagenesis recruits circulating monocytes to the TME [22]. Notably, monocyte-derived macrophages are associated with poorer prognosis compared to microglia [23, 24]. scRNA-seq studies have confirmed the dual ontogeny of TAMs in GBM and revealed competitive dynamics between microglia and macrophages for TME occupancy. Disrupting monocyte migration into the TME has been shown to increase microglial cell presence, underscoring the interplay between these populations [25]. These cells are highly plastic and adopt a continuum of anti-tumoral and pro-tumoral functions, previously described as M1/M2 polarization [26]. Specifically, M2 macrophages have been associated with pro-tumoral functions, such as stimulation of tumor angiogenesis and growth, inhibition of effector T cells, and poor prognosis [19, 27].

The rapid outgrowth of tumor cells leads to an insufficient oxygen supply and hypoxia, which is a major hallmark of therapy resistance to the standard of care in GBM and inhibits anti-tumor immune responses [28, 29]. GBM and immune cells adapt to the hypoxic TME through activation of HIFs; HIF-1α, and HIF-2α being the most studied [30]. The role of HIF-1α has been explored in several solid tumors [31, 32], however, the role of HIF-2α in tumor progression is more limited. HIF-2α drives macrophage polarization towards a pro-tumoral phenotype [33] and its deletion in the myeloid lineage resulted in decreased TAM infiltration and reduced tumor progression in hepatocellular and colitis-associated carcinoma and breast cancer models [34, 35]. In addition, the deletion of HIF-2α in Tregs suppressed tumor growth and melanoma metastasis in mice. Furthermore, the administration of Tregs treated with a selective HIF-2α inhibitor PT2385 prevented the growth of colon adenocarcinoma in vivo [36].

PT2385, a first-in-class orally available HIF-2α inhibitor, was originally developed to treat renal cell carcinoma (RCC) with Von Hippel-Lindau (VHL) mutations. This selective small molecule antagonizes HIF-2 by binding to a structural pocket of the alpha subunit, impeding its dimerization with the aryl hydrocarbon receptor nuclear translocator subunit [37]. This inhibitor was well tolerated and demonstrated clinical activity in RCC [38], and in GBM patients, PT2385 was well tolerated with limited activity and variable drug exposure [39]. Nonetheless, HIF-2α is expressed in GBM, and its expression correlates with decreased OS [40, 41]. Moreover, selective inhibition of HIF-2α using PT2385 extended animal survival in a GBM patient-derived xenograft (PDX) model, although no significant difference in tumor size was observed [41]. Interestingly, in vitro treatment of the PDX cell lines with PT2385 did not impact cell proliferation or viability, suggesting that the drug affected other cells of the TME; however, whether HIF-2α inhibition impacts the immune cells of the GBM tumor microenvironment has not been assessed.

Here, we show that pharmacological inhibition of HIF-2α with PT2385 as single-modality treatment prolonged overall survival and decreased tumor volume in an immunocompetent mouse glioma model. HIF-2α inhibition reshaped the composition of the tumor microenvironment by reducing the proportion of macrophages, increasing the presence of microglia, and promoting their activation, together with a significant decrease in Tregs. This resulted in an attenuation of the immunosuppressive features of the glioma TME, boosting the response to ICB. PT2385 combined with αPD-1 and αTIM-3 therapeutic antibodies resulted in the long-term survival of glioma-bearing mice and a reshaped TME, with enhanced antitumor immunity. Through single-cell RNA sequencing (scRNA-seq) and flow cytometry we observed that the efficacious combinatory therapeutic approach promoted pro-inflammatory macrophages, homeostatic microglia, reduced Treg infiltration and reduced T cell exhaustion.

## Methods

### Cell lines

The mouse glioma cell lines GL261 and SB28 were kindly provided by Dr. Hideo Okada (University of California, San Francisco, CA, USA). The mouse glioma cell line CT-2A was kindly provided by Dr. Thomas Seyfried (Boston College, Boston, MA, USA). The BV2 cell line was kindly provided by Pr. Thierry Soldati (Faculty of Science, University of Geneva). All cell lines were tested negative for mycoplasma contamination by PCR. SB28, GL261, and CT2A were cultured in DMEM-GlutaMAX containing high glucose (4.5 g/l) and 1 mM sodium pyruvate, supplemented with 10% heat-inactivated fetal calf serum, 2.5% HEPES, 1% non-essential amino acids and 1% penicillin/streptomycin (all from Life Technologies).

### Stereotaxic tumor implantation

Tumor cells were implanted orthotopically as previously described [5], using female 6–8-week-old C57BL/6J mice (Charles River Laboratories). Tumor-bearing mice were monitored daily for body weight loss or appearance of symptoms and euthanized according to Cantonal and Federal guidelines. All animal experimental studies were reviewed and approved by institutional and cantonal veterinary authorities in accordance with the Swiss Federal law. For stereotaxic implantation, buprenorphine (Temgesic^®^, RB-Pharmaceuticals) was injected subcutaneously (s.c.) (0.1 mg/kg) one hour before the procedure. Mice were anesthetized with a mixture of ketamine (80 mg/kg, Warner-Lambert) and Rompun: Xylazine (10 mg/kg, Bayer). Using a stereotactic frame (Stoelting), a burr hole was made in the skull 2.6 mm lateral to the right and 1.6 mm rostral to the bregma using a 0.7 mm drill bit and a non-coring needle (Hamilton 7804-04) was used to inject the cells at a depth of 3.6 mm into the brain from the skull. 50,000 GL261 cells were injected in 3 µL HBSS in the pallidum at 3.4 mm into the brain from the skull. The burr hole was covered with bone wax, and the skin incision was sutured. Mice were given buprenorphine (10 mg/ml) in drinking water for 72 h post-surgery.

### Treatment regimens and dosing

**Fig. 1:**
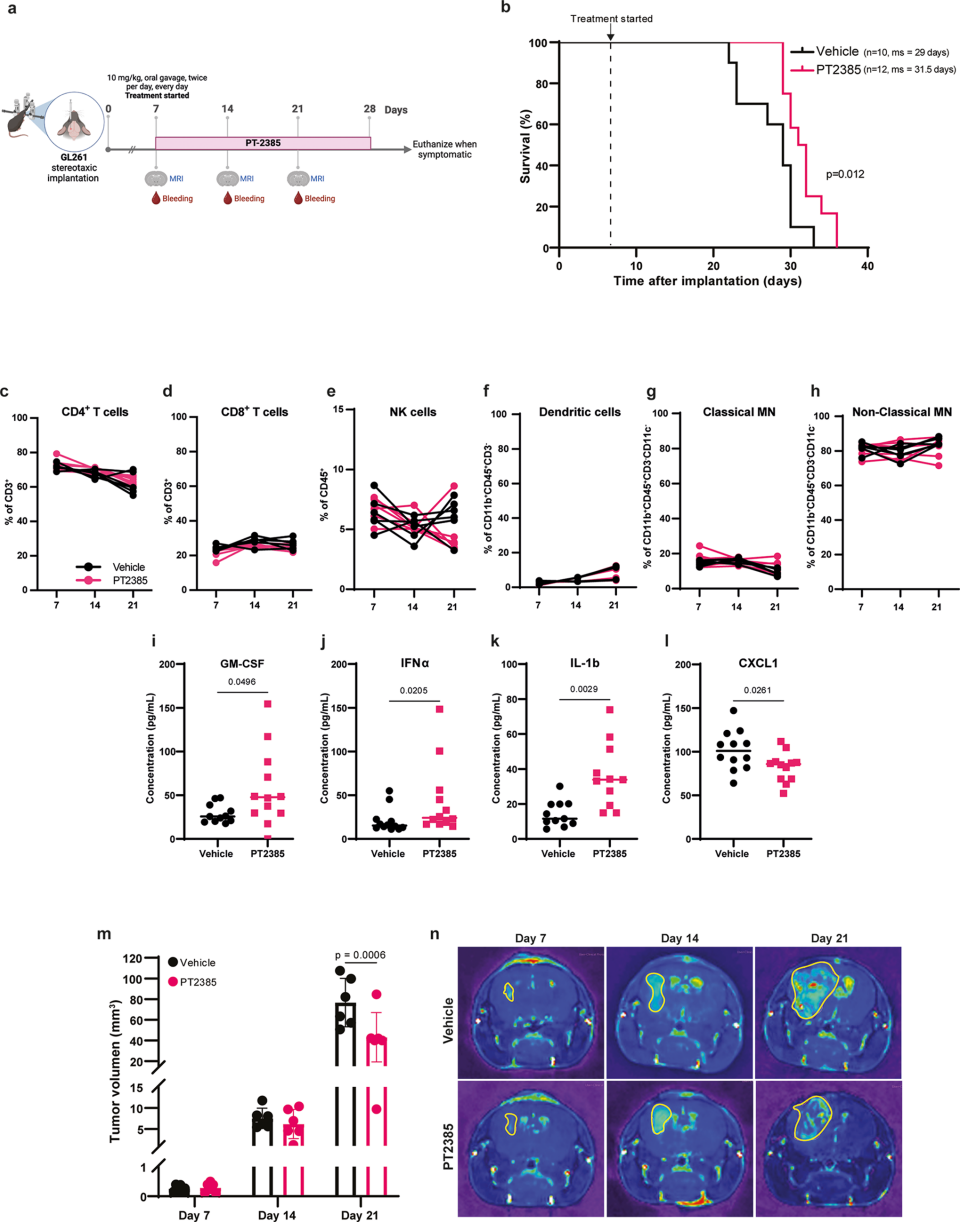
HIF-2α inhibition prolongs survival of GL261-bearing mice and decreases tumor volume. (**a**) Treatment schedule of mice implanted intracranially with GL261 cells. (**b**) Kaplan-Meier survival curve of mice administered with vehicle (*n*=10 females) and PT2385 (*n*=12 females). ms: median survival. Statistical analysis used the Log-rank Mantel-Cox test. (**c**–**h**) Proportions of lymphoid cells (CD4^+^ and CD8^+^ T cells; andCD56^+^CD3^-^ NK cells) and myeloid cells (dendritic cells: CD11c^+^IA-IE^+^; classical monocytes: Ly6C^+^Ly6G^-^ and non-classical monocytes: Ly6C^+^Ly6G^-^) in the peripheral blood of vehicle (black) and PT2385 (pink)-treated mice at days 7, 14 and 21 post-implantation. Graphs show mean ± SD, *n*=6 per group; p-values calculated using a paired t-test. MN: monocytes. (**i**–**l**) Cytokine quantification in plasma collected from peripheral blood at day 21 post-implantation. Graphs show mean ± SD, *n*=12 per group; p-values calculated using an unpaired t-test. (**m**) Tumor volume (mm^3^) of vehicle (black) and PT2385-treated (pink) mice at days 7, 14 and 21 post-implantation. Tumor volume was calculated by the area of sequential coronal plane images. Graph shows mean ± SD, *n*=6 mice per group; p-values calculated using Tukey’s multiple comparison test. (**n**) Representative images of the coronal plane of T1 weighted MRI scans of vehicle and PT2385-treated mice at days 7, 14 and 21 post-implantation

PT2385 (MedChem) was dissolved in 30% PEG400 (Merck), 10% ethanol (Merck), and 60% aqueous solution (0.5% Tween 80 (MedChem) and 0.5% methylcellulose (Sigma-Aldrich)). PT2385 reported a brain-to-plasma ratio of 0.9 in rodents, supporting an efficient blood-brain barrier penetration [37, 41]. PT2385 was administered at 10 mg/kg every day and twice per day in half of the animals; the other half received vehicle. The drug was administered for 21 days from day seven post-implantation. Antibodies were purchased from Bio X Cell. αPD-1 (RPM1-14), αTIM-3 (B8.2C12), rat IgG2a isotype control (2A3), and rat IgG2b isotype control (HRPN) were diluted with sterile PBS and injected intraperitoneally (i.p.) according to doses and treatment schedules displayed in Fig. 1a.

### Magnetic resonance imaging (MRI)

For MRI longitudinal monitoring of tumor growth, mice were injected with gadolinium-based contrast injection (Dotarem^®^, Guerbet-AG) i.p. in saline solution and immediately anesthetized by isoflurane inhalation. Mice were placed on the warm plate in the NanoScan^®^ MRI 3.0 (RS2D). Respiration and temperature were controlled to detect any stress or pain. MRI was performed on days 7, 14, and 21 post-implantation. Images were processed using Horos v3.0 software.

### Isolation of brain infiltrating lymphocytes (BILs)

Mice were sacrificed and perfused with Ringer’s solution (Bischel). Tumor resection samples were enzymatically and mechanically dissociated using the Brain Tumor Dissociation Kit (Miltenyi Biotec) to generate a single-cell suspension according to the manufacturer’s instructions. Myelin debris was removed from the single-cell suspension using the Myelin Removal Beads II (Miltenyi Biotec) according to the manufacturer’s instructions. The cell suspensions were divided for different assays: flow cytometry, RNA extraction, and cytokine measurement.

### Flow cytometry

Cells were incubated with LIVE/DEAD Fixable Dead Cell Stain Kit (ThermoFisher Scientific) in PBS 1X, blocked for Fc receptor binding with anti-mouse CD16/CD32 antibody (clone 2.4G2, BD Pharmigen), then cell-surface stained with optimally titrated antibodies in flow cytometry staining buffer (PBS, 2% FCS, 0.05% sodium azide). Then, cells were permeabilized with the Cytofix/Cytoperm kit (BD Biosciences) and incubated with antibodies diluted in permeabilization buffer. Stained cells were analyzed on a BD LSR-Fortessa (BD Biosciences) or a Gallios flow cytometer (Beckman Coulter), and data were analyzed using Flowjo 10 (BD Biosciences). Gating example of T cells and myeloid cells are schemed in Supplementary Fig. 12a, b. Antibody panels are detailed in Supplementary Table 1.

### Blood collection

Peripheral blood collection was performed on days 7, 14, and 21 post-implantation. Mice were gently warmed with a heat lamp for at least 10 min before the procedure. Mice were placed in a restraining tube and then bled from the tail vein. A density gradient centrifugation was performed using Lymphoprep (StemCell Technologies) to isolate mononuclear cells.

### Multiplex cytokine assay (LEGENDplex)

Cytokine measurement was performed in plasma and brain single-cell suspension using the LEGENDplex™ Mouse Anti-Virus Response Panel 13-plex and the LEGENDplex™ Mouse Macrophage/Microglia Panel 13-plex (BioLegend). The kit was used according to the “LEGENDplex™ Multi-Analyte Flow Assay Kit” instructions. Cytokines were measured with a Gallios flow cytometer and data were analyzed with LEGENDplex™ Data Analysis Software 8.0 (Biolegend).

### BV2 polarization

BV2 mouse microglia cell line was differentiated in vitro with IL-4 (20 ng/ml) and IL-13 (20 ng/ml) (Immunotools) for 24 h for M2 polarization and IFNγ (20 ng/ml) and LPS (100 ng/ml) (Immunotools) for 24 h for M1 polarization.

### qRT-PCR

cDNA was synthesized from 500 ng of total RNA using a mix of random hexamers-oligo d(T) primers and PrimerScript reverse transcriptase enzyme (Takara) following the manufacturer’s instructions. cDNA samples were diluted 5–15 times; primer pairs were pooled and used at 60 nM each, and 14 PCR cycles (95 °C 15 s − 60 °C 4 min) were applied. PCR reactions contained diluted cDNA, 2 x Power SYBR Green Master Mix (Applied Biosystems), and 300 nM of forward and reverse primers. qRT-PCRs were performed on an SDS 7900HT instrument (Applied Biosystems) with the following parameters: 50 °C for two minutes, 95 °C for ten minutes, and 45 cycles of 95 °C 15 s-60 °C one minute. Each reaction was performed in three replicates on a 384-well plate. Raw Ct values obtained with SDS 2.2 (Applied Biosystems) were imported in Excel, and normalization factor and fold changes were calculated using the GeNorm method.

### Cell sorting for bulk RNA sequencing (RNAseq)

Microglia and macrophages were sorted from brain single-cell suspension after cell surface staining in flow cytometry staining buffer. Cells were suspended in Stain buffer (BD Pharmigen) at a density of 1 million per 100uL and stained with anti-mouse CD16/CD32 Fc Block (BD Pharmigen) for 10 min. Next, cell surface antibodies were added, and cells were incubated for 20 min at 4 °C, washed with PBS, and sorted in PBS 1% BSA. Microglia and macrophages were sorted based on CD45^+^Ly6G^−^Ly6C^−^CD11b^+^CD3^−^ gating for both cell populations and then differentiated as CD49d^+^TMEM119^−^DRAQ7^−^ (macrophages) and CD49d^−^TMEM119^+^DRAQ7^−^ (microglia). Sorting was performed on a MoFlo Astrios (Beckman Coulter). Cells were resuspended in RLT buffer (Qiagen) for total RNA extraction and purified using the RNAeasy micro kit (Qiagen). Purification included a DNase treatment using the RNase-free DNase set (Qiagen). All procedures were performed according to the manufacturer’s specifications.

### Bulk RNAseq

cDNA libraries were prepared by the Genomic platform of the University of Geneva as follows: the SMART-Seq mRNA kit (Clontech) was used for the reverse transcription and cDNA amplification according to the manufacturer’s specifications, starting with 1 ng of total RNA as input. 200 pg of cDNA was used for library preparation using the Nextera XT kit (Illumina). Library molarity and quality were assessed using the Qubit (ThermoFisher Scientific) and Tapestation (DNA High sensitivity chip, Agilent Technologies). Libraries were sequenced on a NovaSeq 6000 Illumina sequencer for SR100 reads. Reads were aligned using STAR (v.2.7.0)22 to the mouse GRCm39 reference genome. Differential expression analysis (DEG) was performed using the R/Bioconductor edgeR package. The counts were normalized according to the library size and filtered. Genes with counts above 1 count per million reads in at least three samples were retained for subsequent analysis. Tests for differentially expressed genes were performed using a generalized linear model. DEG was reported with adjusted p-values (Padj) < 0.05 and LFC > 0.5 or LFC < -0.5 with a 5% false-discovery rate (FDR) Benjamini–Hochberg multiple-testing correction. Volcano plots were generated using the EnhancedVolcano package.

### Sorting of CD45^+^ cells by flow cytometry for scRNAseq

From four individual mice from each treatment group, cells were suspended in Stain buffer (BD Pharmigen) at a density of 1 million per 100μL and stained with anti-mouse CD16/CD32 Fc Block (BD Pharmigen) for 10 min. Next, an anti-mouse CD45 antibody (Clone 30-F11, BD Pharmigen) was added, and cells were incubated for 20 min at 4 °C, washed with PBS, and sorted in PBS 1% BSA. Sorting was performed on a MoFlo Astrios (Beckman Coulter). Directly after sorting, cell quantity and viability of CD45^+^DRAQ7- cells were measured, and two pools were prepared from each treatment group comprised of 5000 cells from each of two individual mice.

### sc RNAseq

Tumors were harvested at day 21 p.i. and tumor infiltrating leukocytes were sorted (CD45^+^ DRAQ7^-^). Each cell suspension was loaded on a 10× Genomics Chromium instrument. Single-cell RNA-Seq libraries were prepared using Chromium Single Cell 3′ v3.1 Reagent Kit according to manufacturer’s protocol. Library quantification and quality assessment was performed using a Qubit fluorometer and a Tapestation (DNA High sensitivity chip, Agilent Technologies). Libraries were sequenced on an Illumina NovaSeq 6000 using paired-end 28 × 90 bp as sequencing mode.

### scRNAseq analysis

Raw count matrices were generated using CellRanger 2.2.0 (10x Genomics) with a custom reference package based on mouse reference genome GRCm39 and GENCODE63 gene models. Individual count tables were merged using CellRanger aggr to reduce batch effects. A total of 40,144 cells from all merged samples were analyzed. The average of the mean reads per cell across all gene expression libraries was 41,118, with an average of the median genes per cell of 3083, calculated by Cell Ranger. All scRNAseq data were merged, and the analysis was performed in R using the Seurat v5 package. Data was filtered for potential empty droplets, cell aggregates, and cells with > 2500 or < 200 transcripts were excluded from the analysis. Additionally, low-quality/dying cells with > 5% of transcripts corresponding to mitochondrial genes were excluded. Raw counts were normalized in Seurat by a global-scaling normalization and log-transform method (LogNormalize) that normalizes the gene expression measurements for each cell by the total expression and multiplies it by a scale factor (10,000) and log-transforms the result. High-variable genes were detected in Seurat, and the data were scaled by a linear transformation. Subsequently, the highly variable genes were used for unsupervised dimensionality reduction. Dimensionality was determined using a heuristic method that ranks the principal components using the ElbowPlot function. Differential expression analysis between cell clusters was performed using the Wilcoxon rank-sum test through the Seurat FindMarkers function. Clustering was visualized in UMAP two-dimensional scatter plots using the Seurat v5 package. Plots were generated in R using the scCustomize package. Cells were clusterized through manual annotation of known marker genes, and lymphocyte scRNAseq data were projected into reference mouse TIL single-cell atlas using the ProjectTILs R package [42].

### HIF-2α knock-out

CRISPR/Cas9 technology was used to knock out *Epas1*, the gene encoding HIF-2α, on BV-2 cells. Predesigned CRISPR guide RNA (gRNA) was designed through MISSION ™ gRNA platform (Merck), and Cas9-GFPProtein (Sigma-Aldrich) were transfected using Lipofectamine™ 3000 (ThermoFisher) following the manufacturer’s instructions. Transfected cells were cloned after GFP-positive cell selection through cell sorting.

### Viability assay

Cell viability was assessed on cultured cells in flat-bottom 96-well plates for 48 h under the appropriate conditions and treatment. Viability read-out was performed using Cell Titer Glo (Promega), containing cell lysis buffer and luciferin to allow ATP quantification. Following an incubation of 20 min at RT in the dark, lysates were transferred into flat-bottom white 96-well plates, and the luminescence signal was read within 10 min using a Cytation 3 plate reader (BioTek).

### Migration assay

For the mouse T cell migration assay, polyclonal T cells (CD4^+^ and CD8^+^ T cells) were isolated from C57BL/6J mouse spleens then activated and expanded with CD3/CD28 coated beads (ThermoFischer) at a 1:1 bead/cell ratio and the addition of IL-2 (100 U/ml, Immunotools) and TGF-β (10 ng/ml, PeproTech) on days 0 and 2 for Treg polarization. Tregs pretreated with pertussis toxin (PTX, 100 ng/ml) were used as a negative migration control; for a positive control, mouse CXCL10 (30 ng/ml) and CCL22 (200ng/ml) was added directly to the well. M2 polarized BV2 cells were seeded in 24 well plates 24 h before the co-culture and treated with PT2385 (10 µM). Transwell cell culture inserts (Greiner Bio-One) were placed in each well and 10^5^ Tregs were added in the inserts. After 18 h, the inserts were removed, Tregs (CD4^+^, CD25^+^, FOXP3^+^) were quantified using flow cytometry.

### Statistical analysis

Statistical analysis between groups was performed using a two-tailed unpaired Student’s t test unless otherwise indicated. Kaplan-Meier survival curves were analyzed using the log-rank (Mantel-Cox) test. Data were analyzed using GraphPad Prism v.9. P values less than 0.05 were considered significant unless otherwise indicated.

## Results

### HIF-2α inhibition improves the survival of GL261-bearing mice and decreases tumor volume

To investigate the influence of HIF-2α in glioma progression, we used immunocompetent mice orthotopically implanted with GL261 glioma cells. From day seven post-implantation, we orally administered the HIF-2α inhibitor PT2385 twice daily, for 21 days (Fig. 1a). PT2385 modestly but significantly increased median survival compared to vehicle-administered mice, from 29 to 31.5 days (Fig. 1b). Changes in peripheral immune cell populations were not observed (Fig. 1c-h). Analysis of cytokines at midterm in plasma revealed that PT2385 significantly increased the concentration of IL-1β, IFNα, and GM-CSF (Fig. 1i-k) and significantly decreased the concentration of CXCL1 (Fig. 1l). Additionally, enhanced survival in PT2385-treated mice correlated with reduced tumor volume by day 21 post-implantation compared to vehicle-treated mice (Fig. 1m, n). However, PT2385 did not affect the viability of GL261 cells in vitro, nor of two other mouse glioma cell lines tested (SB28 and CT-2A) (Supplementary Fig. 1). Altogether, these results suggest that PT2385 may affect the tumor microenvironment.

### HIF-2α inhibition impacts infiltration of microglia and macrophages and their transcriptome profile

**Fig. 2:**
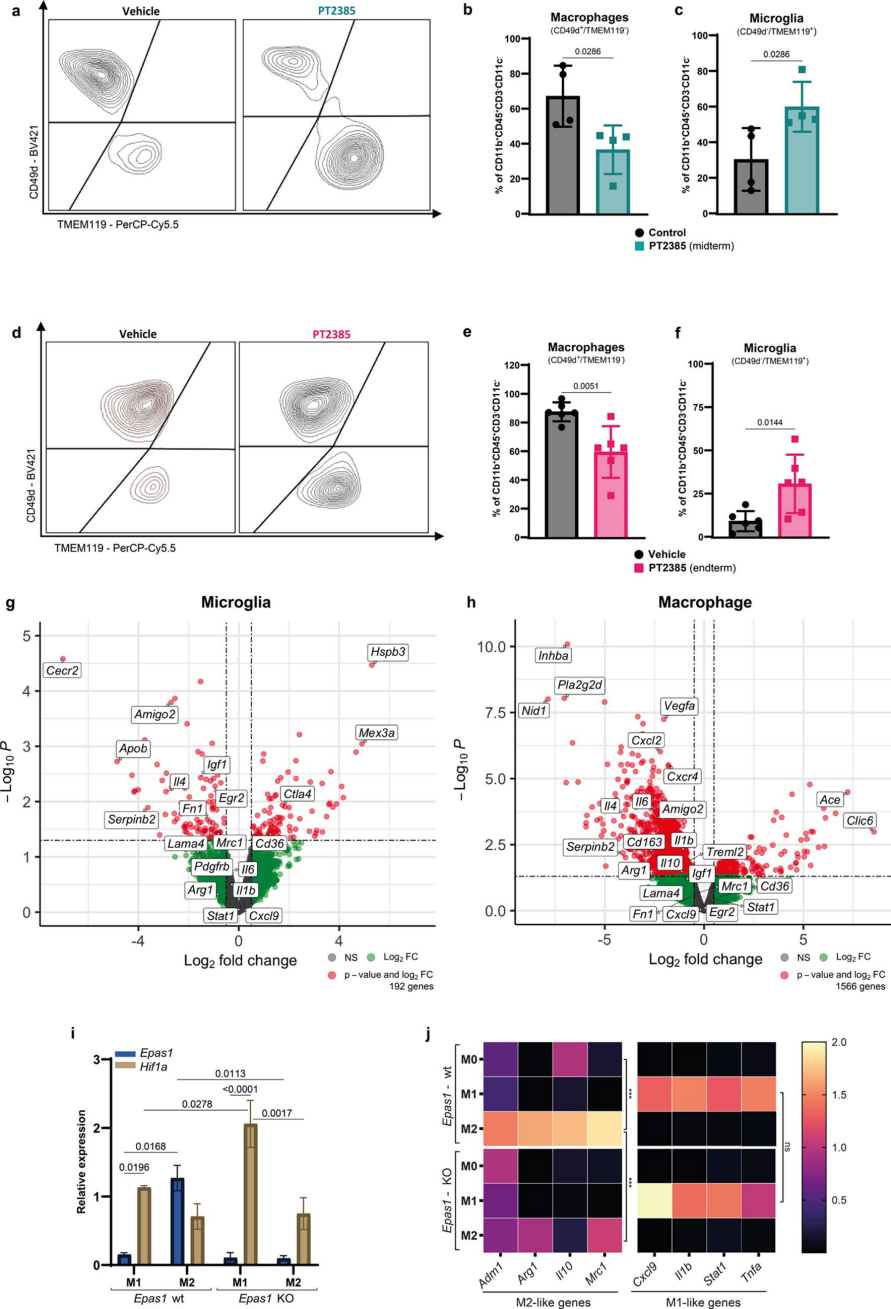
HIF-2α inhibition impacts infiltration and transcriptome signature of microglia and macrophages, and its knockout prevents M2 microglial polarization. (**a**, **d**) Representative flow cytometry plots of macrophages (CD49d⁺TMEM119⁻) and microglia (CD49d⁻TMEM119⁺) gated from live CD45⁺CD11b⁺CD3⁻ cells in vehicle- and PT2385-treated mice. (**b**, **c**) Quantification of macrophage and microglia populations at midterm (green, *n* = 4 per group). (**e**, **f**) Quantification of macrophage and microglia populations at endterm (pink, *n* = 6 per group). Graphs show mean ± SD. p-values were calculated using an unpaired t-test. (**e**–**f**) Vulcano plots of differentially expressed genes (DEG) in sorted microglia and macrophages from vehicle-treated mice versus PT2385-treated mice (Red: Padj < 0.05, LFC > 0.5 or LFC < -0.5, green: LFC > 0.5 or LFC < -0.5 and grey: not significant). (**g**) Quantification of *Epas1* (blue) and *Hif1a* (brown) mRNA by qRT-PCR, normalized to two housekeeping genes (*Gapdh* and *Actb*). The graph shows mean ± SD, *n*=3; statistics used a two-way ANOVA with Tukey’s multiple comparisons test. (**h**) Heatmap of M1- and M2-like genes quantified by qRT-PCR. Graphs show relative expression normalized to two housekeeping genes (*Gapdh* and *Actb*), represented by the median, *n*=3; p-values calculated using a two-way ANOVA with Tukey’s multiple comparisons test

The absence of a major impact of PT2385 at a systemic level suggested a local effect of HIF-2α inhibition in the glioma microenvironment. We hypothesized that HIF-2α inhibition influenced the major subsets of glioma infiltrating immune cells, i.e., macrophages and microglia. Indeed, HIF-2α inhibition impacted the balance of proportions of microglia and macrophages at both mid and endterm time points (Fig. 2a-f). Specifically, PT2385 treated mice showed significantly reduced proportions and numbers of CD49d^+^/TMEM119^−^ macrophages (Fig. 2b, e and Supplementary Fig. 2a, b), with a commensurate increase of CD49d^−^/TMEM119^+^ microglia (Fig. 2c, f and Supplementary Fig. 2c, d). The combined number of microglia and macrophages significantly decreased at both time points upon HIF-2α inhibition (Supplementary Fig. 2e, f), but other myeloid cells, such as dendritic cells, neutrophils, and myeloid-derived suppressor cells (MDSCs) were not affected (Supplementary Fig. 2g-j). Given that HIF-2α inhibition principally impacted the balance between macrophages and microglia, we next performed bulk RNA sequencing of sorted microglia and macrophages at midterm (Supplementary Fig. 3a). First, we validated the ontogeny of the sorted cells; there was a high differential expression of characteristic genes such as *Itga4*, *Itgal*, *Ifitm2*, and *Tgfbi* for macrophages and *Tmem119*, *Sall1*, *Siglech*, and *P2ry12* for microglia (Supplementary Fig. 3b). Then, we evaluated the impact of HIF-2α inhibition on the sorted cell populations. We identified 1566 differentially expressed genes (DEG) in macrophages and 192 DEG in microglia (Fig. 2g, h and Supplementary Table 7). Gene set enrichment analysis (GSEA) in the upregulated and downregulated genes from the DEG showed that both cell populations significantly upregulated the oxidative phosphorylation and the E2F target hallmarks upon HIF-2α inhibition. Conversely, the downregulated genes were related to the hallmarks of TNFA signaling via NFKB, mitotic spindle, inflammatory response, and TGF-β signaling (Supplementary Fig. 3c, d). Notably, there was a significant decrease in the hypoxia and heme metabolism hallmarks only in microglia (Supplementary Fig. 3c). Additionally, we observed an enrichment of lipid metabolism-associated hallmarks solely in macrophages (Supplementary Fig. 3d). Finally, whereas it has been described that HIF-2α regulates M2-like macrophage polarization [33, 34], the extent to which this operates in brain tumors, remains unexplored. To address this, we investigated the differentially expressed genes in both cell populations. Genes known to be associated with an M2-like phenotype, including *Il4*, *Cd163*, *Il10*, *Mrc1*, *Treml2*, *Cxcl2*, *Igf1*, *Egr2*, *Itga10*, and *Il6*, were present in the downregulated gene set of macrophages and microglia, without a concurrent upregulation of genes associated with the M1-like phenotype (Fig. 2g, h). These results indicate that HIF-2α inhibition regulates the balance of glioma-infiltrating microglia and macrophages and potentially their anti-tumor functions.

### HIF-2α knockout (KO) prevents M2 microglia polarization without modifying M1 polarization in vitro

HIF-2α expression has been described to promote M2-like macrophage polarization [33, 34], but its role in microglial cell polarization is unknown. To directly explore the role of HIF-2α in M1/M2 microglia polarization, we deleted the *Epas1* gene encoding HIF-2α in the BV2 mouse microglia cell line and compared polarization of KO with wild-type BV2 cells by treating with LPS/IFNγ or IL-4/IL-13 to induce M1 or M2-like polarization, respectively. In wild-type cells, *Epas1* mRNA was mostly expressed by M2 polarized cells, whereas M1 microglia mostly expressed *Hif1a* mRNA (Fig. 2i). Moreover, M1 microglia expressed *Hif1a* mRNA, which was further increased upon *Epas1* KO, with no detectable expression of *Epas1* (Fig. 2i). We also quantified mRNA expression of key genes associated with each polarization state in the wild-type and HIF-2α deficient cells. Whereas wild-type microglia upregulated genes corresponding to their polarization state, HIF-2α deletion prevented upregulation of M2-associated genes (*Adm1*, *Arg1*, *Il10*, and *Mrc1*) at the mRNA level without altering the expression of M1-associated genes (Fig. 2j).

### HIF-2α Inhibition decreases CD4^+^ Tregs

**Fig. 3:**
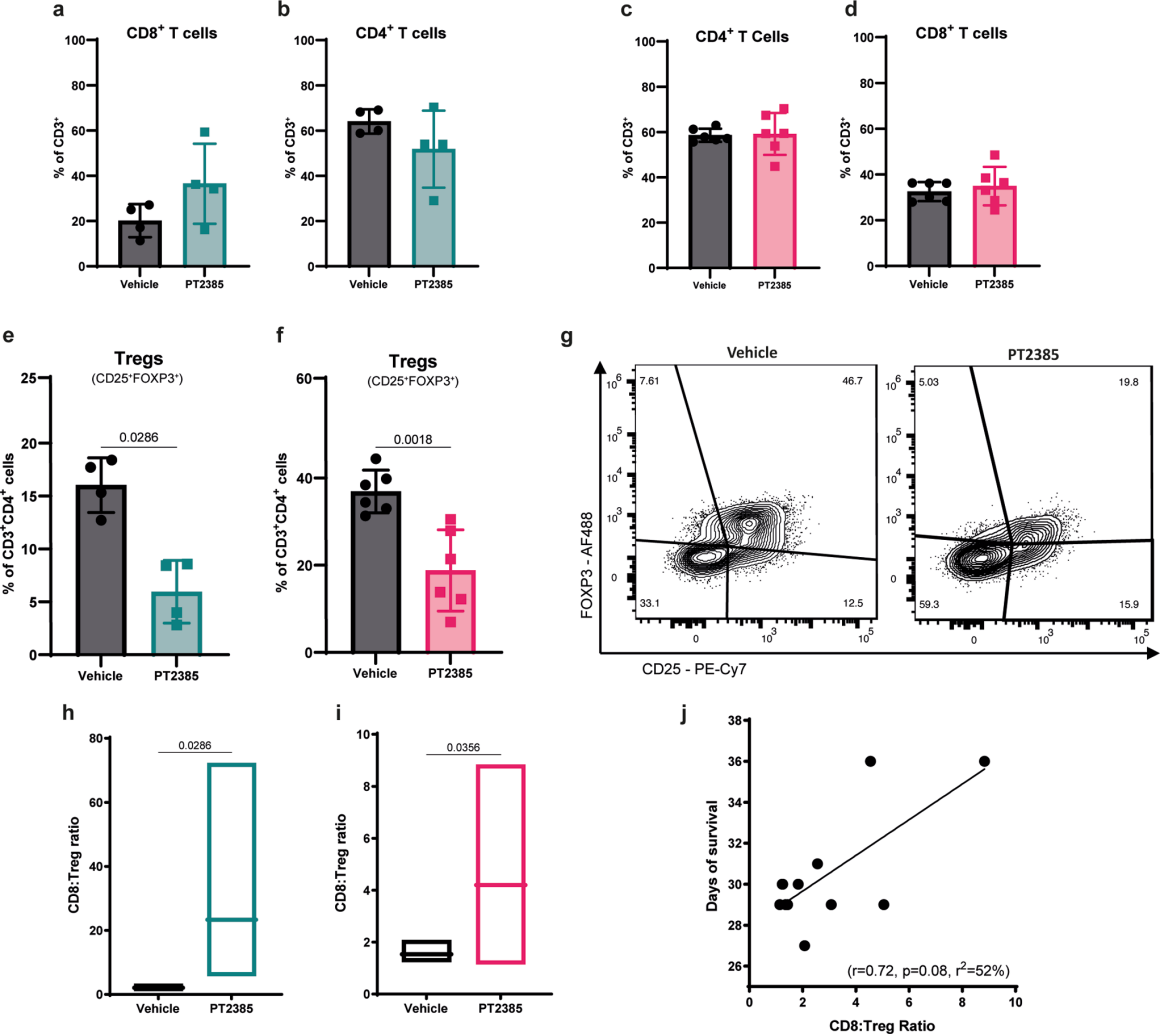
HIF-2α inhibition decreases CD4^+^ Tregs and the CD8:Treg ratio correlates with survival. (**a**–**d**) Flow cytometry quantification of CD8^+^ and CD4^+^ T cells at midterm (green) and endterm (pink). (**e**–**f**) Treg (CD25^+^FOXP3^+^) proportions at midterm and endterm gated on CD4^+^ T cells. (**g**) Representative plot of Treg distribution at endterm of vehicle and PT2385-treated mice. Live cells were gated on CD45^+^CD11b^-^CD3^+^. Graphs show mean ± SD, *n*=4 per group (midterm), *n*=6 per group (endterm); p-values calculated using an unpaired t-test. (**h**, **i**) CD8^+^ T cell: Treg cell ratio. Box plots line show mean, *n*=4 (midterm), *n*=6 (endterm); p-values calculated by an unpaired t-test. (**j**) Pearson correlation between days of survival and the CD8:Treg ratio for each mouse, the black line represents the mean of simple regression

Having characterized the impact of HIF-2α inhibition on myeloid cells, we then studied its effects on glioma-infiltrating T cells through flow cytometry. HIF-2α inhibition did not modify the proportions and numbers of CD4^+^ and CD8^+^ T cells at either midterm or endterm (Fig. 3a-d and Supplementary Fig. 4a-d). However, at both time points, we observed a significant increase in PD-1^+^CD8^+^ T cells (Supplementary Fig. 4g, h), and in the proportion of PD-1^+^TIM-3^+^ CD8^+^ T cells in the PT2385-treated compared to vehicle-treated group at midterm (Supplementary Fig. 4k-m). Interestingly, there were reduced proportions and numbers of CD4^+^ Tregs (CD25^+^FOXP3^+^) at midterm and endterm following HIF-2α inhibition compared to vehicle (Fig. 3e-g and Supplementary Fig. 4e, f). Furthermore, the ratio of CD8^+^ T and Treg cell numbers at both time points was increased in HIF-2α inhibitor-treated mice (Fig. 3h, i). Finally, there was a high and positive correlation between days of survival and the CD8:Treg ratio at endterm (Fig. 3j), suggesting that HIF-2α inhibition mitigated glioma immunosuppression beyond TAMs, specifically impacting the Tregs.

### Synergy of HIF-2α inhibition and ICB to promote long-term survival of GL261-bearing mice

**Fig. 4:**
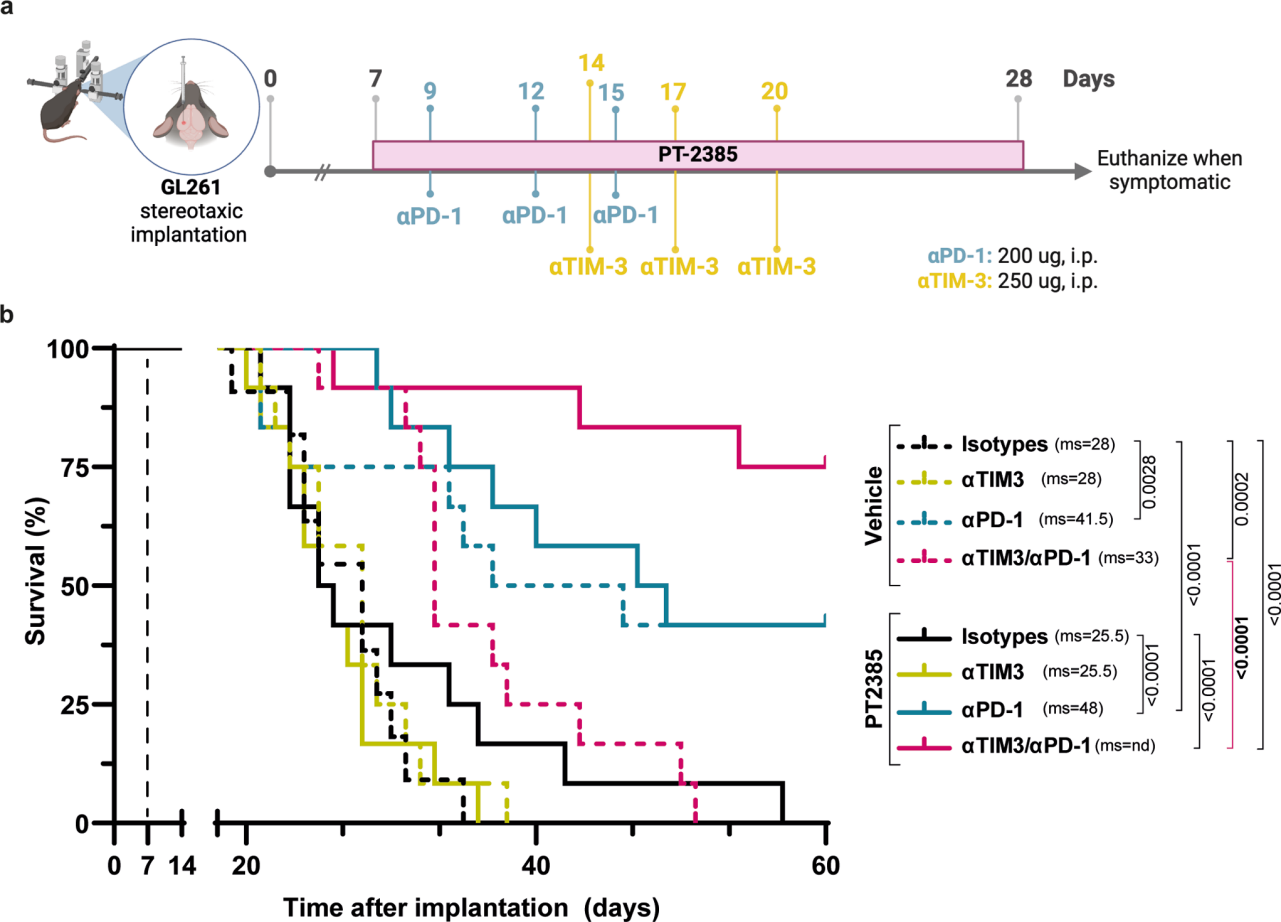
HIF-2α inhibition and ICB act synergistically to promote long-term survival of GL261-bearing mice. (**a**) Treatment schedule of C57BL/6 mice bearing orthotopic GL261 glioma. PT2385 (10 mg/kg) was administered from day 7 post-implantation (p.i.) for 21 days by oral gavage, every day, twice per day. αPD-1 (200 ug) or isotype control antibody or was administered intraperitoneally (i.p.) every 3 days from day 9 p.i. αTIM-3 (250 ug) or isotype control antibody or was administered i.p. every 3 days from day 14 p.i. (**b**) Kaplan-Meier survival curve of mice treated with vehicle (all dashed lines) or PT2385 (all solid lines) in combination with isotype control antibodies (back lines), αTIM-3 alone (yellow lines), αPD-1 alone (blue lines), or in combination with αTIM-3 and αPD-1 (pink lines) antibodies. All mice were females. Sample sizes: vehicle group (*n* = 11), all other groups (*n* = 12). ms: median survival. Comparison of survival curves performed using the Log-rank Mantel-Cox test

Since HIF-2α inhibition only marginally enhanced survival, but alleviated immunosuppressive features of the TME, we hypothesized that PT2385 might enhance ICB efficacy. GL261-bearing mice were randomized into eight groups and treated with PT2385, or vehicle combined with isotype antibodies, αPD-1, αTIM-3, or a mix of the latter (Fig. 4a). Although PT2385 monotherapy did not significantly enhance median survival in this experiment, a minority of mice showed longer survival. Anti-TIM-3 alone or combined with PT2385 did not significantly change median survival compared to the vehicle-treated group or PT2385-treated alone. Conversely, αPD-1, alone (*p* = 0.0028) or combined with PT2385 (*p* < 0.0001), significantly improved the median survival compared to control groups, with a modest trend for increased median survival (41.5 to 48 days) after combined PT2385 and αPD-1 treatment. Strikingly, the combination of dual ICB (αPD-1 plus αTIM-3) with PT2385 dramatically increased survival compared with either PT2385 alone (*p* < 0.0001) or dual ICB alone (*p* < 0.0001) (Fig. 4b). Finally, there was no macroscopic evidence of tumor in asymptomatic mice reaching the day 60 endpoint in mice treated with vehicle plus αPD-1 (2/12), PT2385 plus αPD-1 (3/12), and dual ICB plus PT2385 (9/12), consistent with tumor eradication in these mice.

### Diverse populations of tumor-associated microglia and macrophages infiltrate GL261 gliomas and are differentially modulated by HIF-2α inhibition combined with ICB

**Fig. 5:**
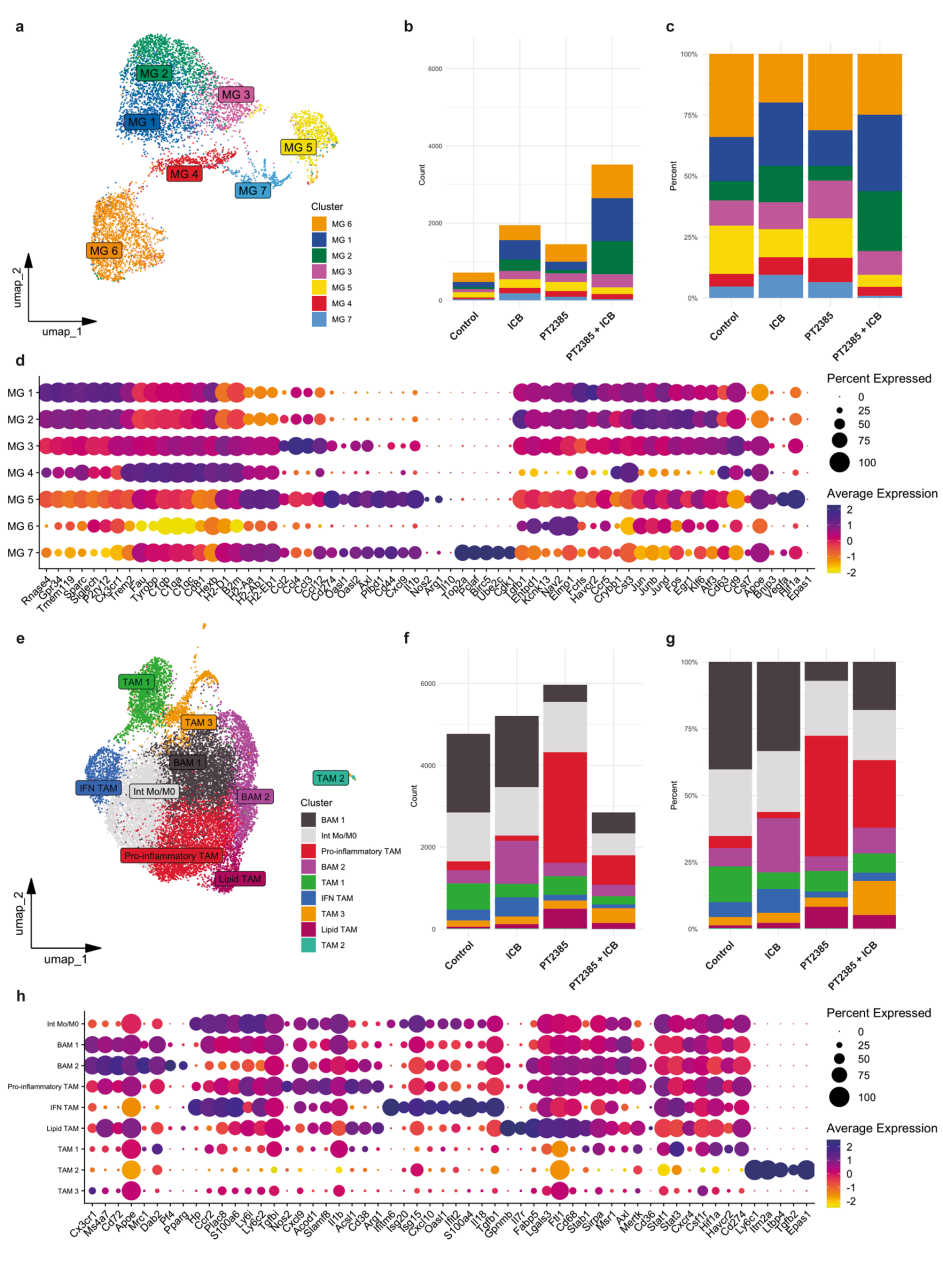
Tumor-associated microglia and macrophages are heterogeneous, and combination therapy impacts their representation within the glioma microenvironment. (**a**) Uniform Manifold approximation and projection (UMAP) plot, (**b**) total counts, (**c**) proportions, and (**d**) dot plot of microglia clusters from GL261-bearing brains at day 21 post-implantation, following unsupervised clustering and manual annotation. (**e**) UMAP plot, (**f**) total counts, (**g**) proportions, and (**h**) dot plot representation of macrophage clusters from GL261-bearing brains at day 21 post-implantation, following unsupervised clustering and manual annotation. Dot plots show average gene expression in gradient color code and percentage of cells expressing the gene represented by circle diameter. For panels b, c, f, and g, counts and proportions of all microglia clusters in each combinatory treatment group (from left to right: control, ICB, PT2385, and PT2385+ICB)

To explore the immune infiltrate of the GL261 TME and pinpoint the immune cells impacted by HIF-2α inhibition, we conducted scRNAseq on CD45^+^ cells sorted from GL261-bearing mice treated with PT2385 and dual ICB (or their respective controls) at day 21 post-implantation (Supplementary Fig. 5a). Through unsupervised clustering, we identified 21 distinct clusters of cells representing diverse populations of macrophages, microglia, T cells, dendritic cells, B cells, NK cells, basophils, and neutrophils (Supplementary Fig. 6b, 7b and Supplementary Table 2). These were classified based on the expression of known marker genes and visualized using heatmaps (Supplementary Fig. 5c) and feature plots (Supplementary Fig. 6a). In all conditions, microglia and macrophage clusters constituted the predominant cell population (~ 75%) among CD45^+^ cells, followed by T cells (~ 17%), dendritic cells (~ 4%), together with a smaller representation of B cells (~ 1.5%), basophils (~ 1%), and neutrophils (~ 1%). (Supplementary Fig. 6c and Supplementary Table 6). To delve deeper into TAM heterogeneity, we reclustered the microglia and macrophage-identified clusters (Supplementary Fig. 7a), unveiling sixteen major TAM clusters (Fig. 5a, e, Supplementary Fig. 7a and Supplementary Table 5). These clusters showed different expression levels of genes associated with either macrophages or microglia [25, 43–45].

When we analyzed the treatment groups separately, the total number of microglia increased in all treatment groups compared to the control group (Fig. 5b). Conversely, compared to the control, macrophage numbers decreased when PT2385 was combined with ICB (Fig. 5f). Notably, this combination significantly increased the number of microglia, surpassing the total number of macrophages (Fig. 5b, f), highlighting a shift in the balance of the TAM composition within the glioma microenvironment. In this condition, the increase in microglia numbers was primarily due to a rise in the homeostatic microglia clusters MG1 and MG2, along with the MG6 cluster (Fig. 5b, d). Moreover, these three clusters represented the highest proportions (> 75%) of all microglia clusters, specifically in mice treated with the combinatory therapy (Fig. 5c and Supplementary Table 6). MG1 and MG2 exhibit elevated expression of canonical microglia-enriched genes *Rnase4*, *Gpr34*, *Tmem119*, *Sparc*, *Siglech*, and *P2ry12* that reflect a homeostatic state (Fig. 5d). MG6 has lower expression of most of the homeostatic genes except for *Nav2*, *P2ry12*, and *Siglech*, but high expression of *Endpd1* and *Kcnk13* (Fig. 5d).

Macrophage reduction was mainly due to decreased numbers of a border-associated macrophage (BAM) cluster BAM 1, characterized by high expression of *Ms4a7*, *Cd72*, *Apoe*, and *Dab2*), the Int Mo/M0 cluster (expressing high levels of *Hp*, *Ccr2*, *Plac8*, *Ly6i*, *Ly6c*, and *Tgfbi*), and the TAM 1 cluster. This latter cluster was one of the three macrophage clusters without upregulation of genes for a specific ontology or function, but TAM 1 cells were distinguished by upregulation of *Hif1a* (Fig. 5f, h). Additionally, we observed a decrease in the proportions of these clusters after combinatory treatment (Fig. 5g). In contrast, the pro-inflammatory cluster (characterized by high expression of *Nos2*, *Cxcl9*, *Acod1*, *Slamf8*, *Acsl1*, *Il1b*, and *Cd38*) increased in numbers and proportions in the groups treated with PT2385 compared to the control group (Fig. 5f, g, h). Two other TAM clusters were modulated after PT2385 treatment: there was a decrease in the IFN TAM cluster (highly expressing type I IFN response associated genes *Ifitm6*, *Isg20*, *Cxcl10*, *Oasl1*, *Ifit2*, and *S100a4*), and an increase in the Lipid TAM cluster that highly expresses lipid metabolism-associated genes *Gpnmb*, *Il7r*, *Fabp5*, *Lgals3*, *Ftl1*, *Cd68*, and *Stab1* (Fig. 5f, g, h). Overall, these results indicate that HIF-2α inhibition remodeled the myeloid immune status in GL261, by favoring the presence of homeostatic microglia and decreasing the presence of pro-tumoral macrophages.

### Heterogenous T cells infiltrate GL261 glioma

**Fig. 6:**
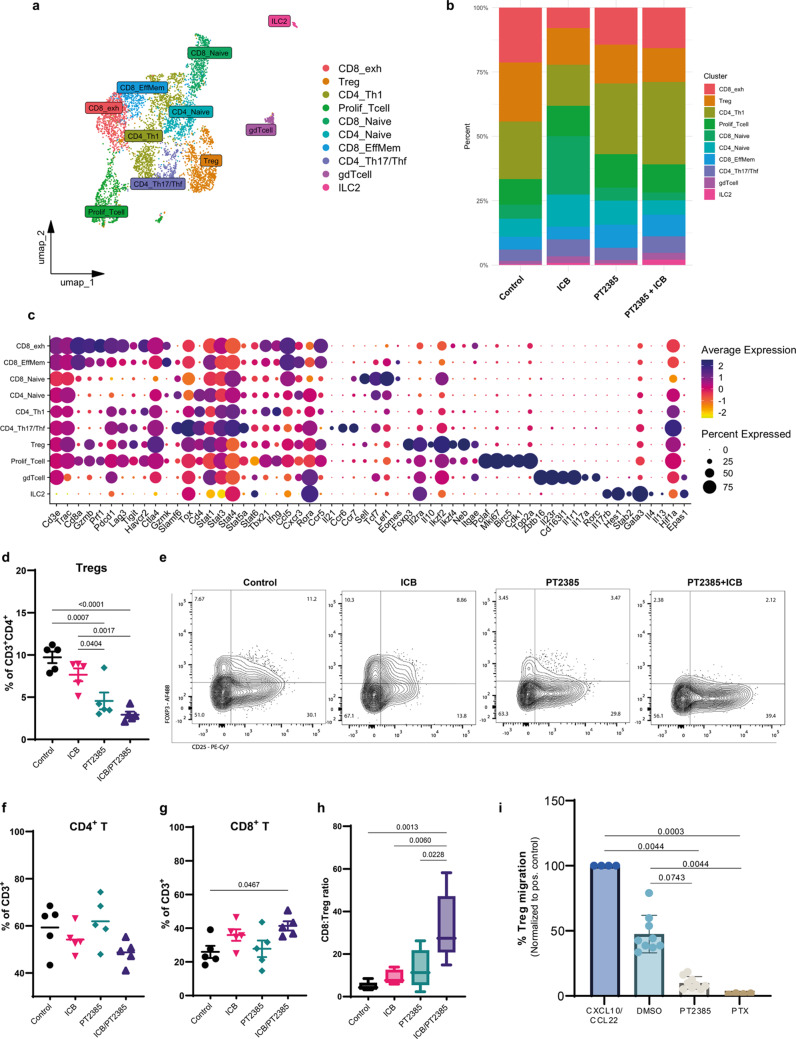
Heterogenous T cell subpopulations infiltrate the glioma microenvironment, and HIF-2α inhibition combined with ICB impacts their representation (**a**) UMAP projection of T cell clusters from GL261 glioma-bearing brains at day 21 post-implantation, showing unsupervised clustering and manual annotation of subpopulations. (**b**) Proportional representation of T cell clusters across treatment groups (vehicle, ICB, PT2385, and ICB/PT2385).(**c**) Dot plot illustrating average expression and percent expression of key markers across T cell subpopulations. (**d**) Proportion of Tregs (CD25^+^FOXP3^+^), gated on CD4^+^ T cells, in each treatment group. Data are shown as mean ± SD, with *n* = 5 mice per group. Statistical significance was assessed using a one-way ANOVA test. (**e**) Representative flow cytometry plots of Treg gating in all treatment groups. (**f**) Proportions of CD4^+^ T cells and (**g**) CD8^+^ T cells, gated on live CD45^+^CD11b^-^CD3^+^ cells in each treatment group. Data are shown as mean ± SD, *n* = 5. (**h**) Ratio between CD8^+^ T cells to Tregs (CD4^+^CD25^+^FOXP3^+^) in each treatment group. Data are shown as box plots (mean ± SD, *n*=5); statistics by one-way ANOVA test. (**i**) Percentage of Treg (CD4^+^CD25^+^FOXP3^+^) migration from top chamber in a transwell assay towards the bottom chamber seeded with M2 polarized BV2 microglia pretreated with PT2385 or DMSO. PTX: Treg pretreated with pertussis toxin as negative control. Treg treated with CXCL10 and CCL22 as positive control. Data normalized by percentage of positive control are shown as mean ± SD, from 2 independent experiments with 4-5 technical replicates per group); statistics by one-way ANOVA test

To explore the impact of the combination of HIF-2α inhibition and ICB in the T-cell compartment, we reclustered the T-cell-identified populations (Supplementary Fig. 8a and Supplementary Tables 3 and 4). Subsequently, we defined the T-cell cluster through unsupervised clustering and manual annotation based on known marker genes (Fig. 6a). To deepen our T-cell cluster definition, we undertook a parallel analysis by projecting our scRNAseq data onto a stable reference single-cell atlas of tumor-infiltrating lymphocytes (TILs), referred as the ProjectTILs method [42](Supplementary Fig. 9). Through both methods, we identified naive-like CD8^+^ T cells, effector memory-like CD8^+^ T cells and exhausted-like CD8^+^ T cells (Fig. 6a, c). Among the clusters identified as CD4^+^ T cells, we identified T helper 1 (Th1) cells, a naïve-like CD4^+^ cluster, a cluster showing a shared signature of both T follicular helper cells (Thf) and Th17 cells, and a Treg cluster (Fig. 6a, c and Supplementary Fig. 9). Additionally, only through manual annotation, we identified a T-cell cluster characterized by a proliferative gene signature (*Pclaf*, *Mki67*, *Cdk1*, *Birc5*, and *Top2a*), which consisted of a mixed T cell population based on the expression of *CD8a*, *Cd4*, and *Foxp3* (Fig. 6c), identified as exhausted CD8 T cell (CD8_Tex) and Treg clusters through the ProjectTILs method (Supplementary Fig. 8a). Furthermore, also only through manual annotation, we identified two small clusters corresponding to γδ T cells, and type-2 innate lymphoid cells (ILC2); both clusters were not included in the atlas-based annotation (Supplementary Fig. 9a).

### HIF-2α inhibition combined with ICB decreased Treg and increased CD8^+^ T cell proportions

CD4_Th1 cluster proportions were increased in mice treated with PT2385 (27.6% alone, 32.0% with ICB) compared to the control group (22.4%) (Fig. 6b and Supplementary Table 6). Moreover, the cluster identified as progenitor-exhausted CD8 T cells (CD8_Tpex) through the ProjectTILs method represented a small proportion of all T cells. However, treatment with PT2385 alone doubled the proportions (8.1%) compared to all groups (~ 3.6%) (Supplementary Fig. 9b and Supplementary Table 6). Flow cytometry confirmed a significant increase of progenitor-exhausted CD8^+^ T cells (SLAMF6^+^TIM-3^−^)(Supplementary Fig. 10f, g). Likewise, the γδ T cell cluster represented a small fraction of total T cells, accounting for 1% of all T cells in the control group, but it increased when PT2385 was combined with ICB to 2.7% (Fig. 6b). Interestingly, through both clusterization methods, we observed a reduced proportions of the Treg cluster in both groups treated with PT2385 (15% alone, 13% with ICB) compared to the control group (23%) (Fig. 6b and Supplementary Table 6). Flow cytometry analysis at day 21 showed that dual ICB combined with HIF-2α inhibitor significantly increased proportions of CD8^+^ T cells (Fig. 6g), without affecting CD4^+^ T cells (Fig. 6f). As observed in the scRNAseq data, HIF-2α inhibition, alone or combined with ICB, led to a significant reduction in the proportions of total Tregs and Granzyme B^+^ Tregs compared to both the control and ICB-treated groups (Fig. 6d, e and Supplementary Fig. 10d, e). Moreover, combination treatment significantly decreased the expression of immunosuppressive molecules such as *Gzmb*, *Il10*, *H2-Ab1*, and *Il2ra*, compared to the control group (Supplementary Fig. 11a-e). Although our analysis did not reveal statistically significant variations in total T cell number across any of the groups (Supplementary Fig. 10a-c), the CD8^+^ T cell to Treg cell ratio was significantly elevated within the group receiving combined ICB and PT2385 therapy, higher than the ratios observed in all other groups (Fig. 6h). Additionally, to gain deeper insights into the cellular mechanisms affected by HIF-2 inhibition, we evaluated the impact of PT2385 on Tregs in vitro using flow cytometry. Our analysis revealed that PT2385 did not affect Treg polarization in vitro (data not shown). Nonetheless, in a transwell migration assay, M2 polarized microglia treated with PT2385 significantly decreased the migration of Tregs in vitro compared with DMSO-treated M2 microglia and with Tregs treated with CXCL10 and CCL22 as a positive control for migration (Fig. 6i).

## Discussion

Here we show that treating GL261-bearing mice with the HIF-2α inhibitor PT2385 profoundly reshaped the immune infiltrate, with major changes in myeloid cells and Tregs. PT2385 changed the balance of glioma infiltrating microglia and macrophages and also their transcriptional profile, with promotion of homeostatic microglia and reduction in pro-tumoral macrophages. The balance of Tregs and CD8^+^ T cells was also altered in favor of CD8^+^ T cells. These changes correlated with a reduction in tumor size and extended survival, particularly in combination with αPD-1 and αTIM-3 based ICB. HIF-2α targeting is clinically efficacious in certain solid tumors, with early studies suggesting promise for GBM [41, 46]. However, the biological consequences of HIF-2α inhibition remain poorly understood. The development of new therapeutic strategies for GBM will likely employ a multifaceted approach built on a comprehension of how the glioma microenvironment is impacted and how the balance of pro- and anti-glioma immune cells can be modulated. In this regard, the potential for HIF-2α inhibition to sensitize to ICB is highly pertinent, since human GBM is generally unresponsive to this therapeutic modality.

We observed that HIF-2α inhibition with PT2385 extended survival in an immunocompetent mouse glioma model. A previous study reported similar survival outcomes in a PDX glioma model using immunodeficient mice [41]. It is noteworthy that PT2385 was reported to operate through a cytostatic mechanism rather than a cytotoxic one [47], indicating that tumor reduction via tumor cell killing is not anticipated. We confirmed that PT2385 did not impact the viability of mouse glioma cell lines in vitro, as previously observed for human GBM and RCC cell lines [41, 47], although we observed a favorable reduction in tumor volume after treatment in vivo. Our results are consistent with PT2385 controlling GL261 growth indirectly through the action of cytotoxic and/or phagocytic immune cells recruited to the TME. Indeed, monocytes/macrophages, neutrophils, and Tregs can be recruited to the tumor via CXCL1 and CCL5 chemokines, the genes for which contain hypoxia-response element sequences [48, 49].

We used PT2385 in combination with sequential administration of PD-1 blockade followed by TIM-3 blockade, a regimen proposed to overcome adaptive resistance by TIM-3 upregulation following αPD-1 therapy [50] and reported to be efficacious in lung adenocarcinoma models [51]. In GL261, TIM-3 expression by T cells was reported to increase from day 14 post-implantation, supporting the approach of targeting both checkpoint inhibitors in GL261 as well as in other glioma models [9, 10, 52]. Indeed, we did not observe a therapeutic effect of targeting TIM-3 alone, and adding PT2385 did not alter the outcome. However, combining HIF-2α inhibition with the dual targeting of TIM-3 and PD-1 dramatically increased survival, highlighting the interest in targeting multiple non-redundant axes. We thus hypothesize that combining PT2385 with ICB takes advantage of targeting non-overlapping pathways to potentiate anti-tumor immunity, with PD-1 and TIM-3 blockade primarily reactivating T cell function, while HIF-2α inhibition mitigates other immunosuppressive mechanisms mediated by hypoxia, TAMs, or Tregs [13, 36, 41].

In this regard, the role of HIF-2α on TAMs in the context of glioma has not been reported. Here, we show through scRNAseq and flow cytometry analysis that HIF-2α inhibition results in a decrease in macrophages and an increase in microglia within the TME. It was previously reported that immunostimulatory treatments alter the dynamics between macrophages and microglia, with both cell populations competing for the TME space [5, 25, 53]. Similar to these studies, we associated the shift in TAM proportions to an enhanced anti-tumor response, highlighting macrophages’ pro-tumoral role but not that of microglia. We propose that this proportion change is consistent with a direct action of the HIF-2α inhibitor on monocytes, macrophages, and microglia. In this sense, we observed that PT2385 combined with ICB decreased the Int Mo-M0 cluster representing monocytes infiltrating glioma from the circulation [43]. In vitro studies indicate that TAM HIF-2α may play a role in enhancing recruitment capacity. BMDMs deficient in HIF-2α exhibited decreased migration and invasion in response to CSF-1 and CXCR4 [34]. Similarly, CSF-1 and IL-6 promoted expression of HIF-2α, but not HIF-1α, in macrophages under normoxic conditions [54]. These findings suggest that since HIF-2α can promote macrophage infiltration into glioma, its inhibition can serve as a first step to remodel the TME to abrogate tumor growth and potentiate the action of immunotherapies. The augmented proportions and numbers of microglia within the TAMs after HIF-2α inhibition and their favorable correlation with survival are consistent with data from human GBM, in which activated and homeostatic microglia were associated with improved survival [43].

Other potentially favorable modifications of TAMs induced by HIF-2α inhibition include a lipid-enriched signature, which has been associated with phagocytic functions [25, 55]. Indeed, basal levels of HIF-2α expression in macrophages under normoxic conditions were reported to suppress phagocytosis [56]. Our data is consistent with a model in which HIF-2α inhibition promotes phagocytosis, while dual ICB re-invigorates exhausted T cells, favoring the possibilities for TAMs to present or cross-present tumor-derived antigens to CD4^+^ and CD8^+^ T cells and promote local T-cell re-activation and tumor control [12, 57].

HIF-2α expression has been reported to promote pro-tumoral macrophage polarization [33, 34]. Here, we observed a decreased pro-tumoral profile without a corresponding upregulation in the anti-tumoral profile in both TAMs and microglia following HIF-2α inhibition. A pro-tumoral state in TAMs has been proposed to occur through endothelial cell-derived IL-6 and CSF-1, which promotes the expression of ARG1 and pro-tumoral polarization through the upregulation of HIF-2α, which is induced by peroxisome proliferator-activated receptor γ (PPARγ) [54]. Our study shows that HIF-2α was preferentially expressed in the pro-tumoral microglia compared to their anti-tumoral counterpart. Moreover, the knockout of HIF-2α in a microglial cell line prevented the expression of pro-tumoral genes without affecting the expression of anti-tumoral genes. We suggest that microglial polarization under HIF-2α regulation may mirror the mechanism previously identified in macrophages, where HIF-2α fosters an anti-inflammatory state through ARG1 expression [33, 54]. ARG1 expression promotes L-arginine degradation, decreasing T-cell proliferation, reduced cytokine production, and impaired cytotoxicity [58]. Although ARG1 is used as a typical pro-tumoral marker gene, the simplistic convention of M1/M2 states is mostly applicable to in vitro experiments, whereas TAMs exist in a full spectrum of phenotypes and functions shaped by the nuances of the tumor microenvironment [59, 60]. Our observations, through scRNAseq, support that within the glioma microenvironment, both microglia and macrophages exhibit a continuum of polarization states that extends beyond the M1/M2 dichotomy. Overall, our data suggests that reducing macrophage infiltration and re-educating both microglia and macrophage populations towards an anti-tumoral phenotype through HIF-2α inhibition represents a rational strategy that can impact both innate and adaptive anti-tumor immunity in GBM.

Importantly, modulation of HIF-2α did not only affect the myeloid compartment but also T cells. Specifically, HIF-2α inhibition combined with ICB promoted Th1 CD4^+^ T cells that can favor activation and phagocytic functions of microglia and macrophages through IFNγ, also reported for GBM in the context of CTLA-4 blockade [12]. HIF-2α inhibition also resulted in a two-fold increase in the progenitor-exhausted CD8 cluster, suggesting that HIF-2α inhibition could restore the functionality of exhausted CD8^+^ T cells, with a potential involvement of the TCF-1 axis, as has been previously suggested [61]. Additionally, a fraction of the terminally exhausted CD8 cluster upregulated proliferation-associated genes when HIF-2α inhibition was combined with ICB, consistent with re-invigoration of these cells.

HIF-2α inhibition not only impacts conventional T cells but also influences the γδ T cell populations within the TME. γδ T cells are a rare subset of T cells with the potential to exert anti-tumor functions in an MHC-independent manner through IFNγ, perforin, and granzyme B release [62], with anti-glioma activity demonstrated in vitro and in vivo [63, 64]. Moreover, γδ T cell infiltration correlates with survival in patients with high-grade glioma [65]. Nevertheless, hypoxia was demonstrated to induce loss of functions and apoptosis in tumor-infiltrating γδ T cells in GL261-bearing mice [66], although alleviating tumor hypoxia could restore functionality [65]. Our results support these observations, with the therapeutically efficacious combination of ICB with HIF-2α inhibition favoring the presence of intratumoral γδ T cells. Of note, this treatment combination increased IL-18 concentration in the TME, essential for γδ T cell proliferation and cytotoxicity [62].

Glioma infiltrating Tregs contribute to resistance against immunotherapies, and their abundance negatively correlates with prognosis [17, 18]. In our study, targeting HIF-2α, either as monotherapy or combined with ICB, reduced Treg infiltration and extended survival. It has been described that ablation of HIF-2α in Tregs, but not HIF-1α, impaired the in vivo suppressive function of Tregs. This was linked to a compensatory increase in HIF-1α levels that led to an upregulation of IL-17 and IFNγ, suggesting a conversion of these cells into effector CD4^+^ T cells [36]. Moreover, HIF-2α KO in Tregs did not impact their development or in vitro suppressive activity. In line with this, in our study, HIF-2 inhibition did not affect Treg polarization in vitro. Nonetheless, Tregs co-cultured with M2-like microglia treated with HIF-2 inhibitor prevented Treg migration towards the microglial cells. These findings suggest that the reduction in Tregs observed in vivo may not be due to a direct effect of PT2385 on these cells. Instead, we hypothesize that HIF-2 inhibition is primarily mediated through its effects on microglia and potentially macrophages, which indirectly influence aspects of the immunosuppressive TME, such as Treg infiltration. HIF-2α inhibition may regulate the recruitment of Tregs to the glioma site through a reduction of Treg-attracting chemokines such as CCL17, CCL22, and CCL18 or a re-polarization of the pro-tumoral macrophages secreting these factors [67]. Similarly, TGF-β, which is secreted by M2 macrophages in the TME and is necessary for Treg development and function, may be reduced following HIF-2α inhibition [68]. The significance of the Treg component of the glioma microenvironment will depend upon the proportions of cells with anti-tumor functions. This is illustrated by our observation that a high CD8:Treg ratio was achieved after HIF-2α inhibition alone and with ICB, which positively correlated with longer survival. These results underpin HIF-2α as a critical regulator of the anti-tumor immune response, suggesting HIF-2α blockade as a rational means to mitigate features of the immunosuppressive GBM microenvironment that complements and potentiates responsiveness to immunotherapies such as ICB [69].

Although we focused here on understanding the effects of HIF-2α inhibition within the immune compartment, we do not reject a direct role of PT2385 on glioma cells located in hypoxic zones, where HIF-2α may modulate malignancy, stemness, and production of chemokines that regulate immune cell infiltration and activation [70]. Moreover, an effect on stromal cells, such as endothelial cells and fibroblasts, is expected, further regulating immunosuppression in the TME [13, 71].

No studies have yet evaluated the combination of HIF-2α inhibition with PD-1 and TIM-3 blockade. However, recent findings demonstrated that combining a HIF-2α inhibitor with anti-CTLA-4 significantly slowed tumor growth in a pancreatic adenocarcinoma mouse model compared to anti-CTLA-4 alone, with further addition of anti-PD-1 extending median survival compared to vehicle or HIF-2α inhibitor alone, though not significantly beyond dual ICB therapy [13]. While the mechanisms underlying the synergy between ICB and HIF-2α inhibition remain unclear, our findings align with Garcia et al., supporting this combinatorial approach. We hypothesize that HIF-2α inhibition complements ICB by targeting distinct immunosuppressive pathways, reducing TAM and Treg infiltration while reinvigorating exhausted T cells via PD-1 and TIM-3 blockade.

Although PT2385 showed promising preclinical and clinical anti-tumor activity [37, 38, 41, 47], its efficacy in GBM patients was limited due to variable drug exposure [39] and the acquisition of drug resistance with prolonged use [72]. This limited exposure was attributed to the formation of a secondary metabolite that does not effectively bind to HIF-2 [73]. To address these limitations, belzutifan, a second-generation HIF-2α inhibitor, was developed. In a phase I clinical trial, belzutifan, administered once daily at a dose seven times lower than PT2385, showed a favorable safety profile and encouraging anti-tumor effects [74]. This underscores the necessity for further research to understand the efficacy and mechanisms of HIF-2α inhibitors.

Currently, six ongoing clinical trials are investigating the safety and efficacy of HIF-2α inhibition in combination with ICB (pembrolizumab) for advanced ccRCC or solid tumors (NCT04976634, NCT05239728, NCT05030506, NCT05899049, NCT04736706, and NCT04895748). Considering the encouraging potential of HIF-2α inhibition as a therapeutic strategy, additional studies are crucial to explore whether this approach could benefit other cancer types, such as gliomas.

Our study represents the first detailed investigation into the role of HIF-2α in the immune TME of glioma. We have identified that targeting HIF-2α modulates the proportions of infiltrating TAMs and Tregs, known for their immunosuppressive functions. While ICB has demonstrated efficacy in some mouse glioma models [5, 9–13], its efficacy in human gliomas remains limited [3, 6, 7], highlighting the need for novel therapeutic strategies. Here, we show that in an immunogenic mouse model, HIF-2α inhibition enhances the efficacy of ICB comprised of αPD-1 and αTIM-3, emphasizing the value of combining therapies targeting distinct pathways to overcome glioma progression. These findings lay the groundwork for future research to unravel the precise mechanisms by which HIF-2α influences immune cell function in the glioma TME. Furthermore, our data suggest that HIF-2α targeting, particularly in combination with ICB, could be expanded as a strategy to regulate immunosuppression across various solid tumors, providing a promising approach to enhance anti-tumor immune responses in glioma.

## Electronic supplementary material

Below is the link to the electronic supplementary material.

Supplementary Material 1

Supplementary Material 2

Supplementary Material 3

Supplementary Material 4

Supplementary Material 5

Supplementary Material 6

Supplementary Material 7

Supplementary Material 8

## Data Availability

All datasets from bulk and single-cell RNA sequencing will be made available in the open-access archive Gene Expression Omnibus (GEO) repository.
