# Supplementary material for: Targeting HIF-2α in glioblastoma reshapes the immune infiltrate and enhances response to immune checkpoint blockade
Source: Cell Mol Life Sci. 2025 Mar 17;82(1):119. doi: 10.1007/s00018-025-05642-8 (PMC11914682; doi:10.1007/s00018-025-05642-8)
Supplement: Supplementary file 1 — Supplementary Material 1 [file 18_2025_5642_MOESM1_ESM.docx]

# Supplementary Figures


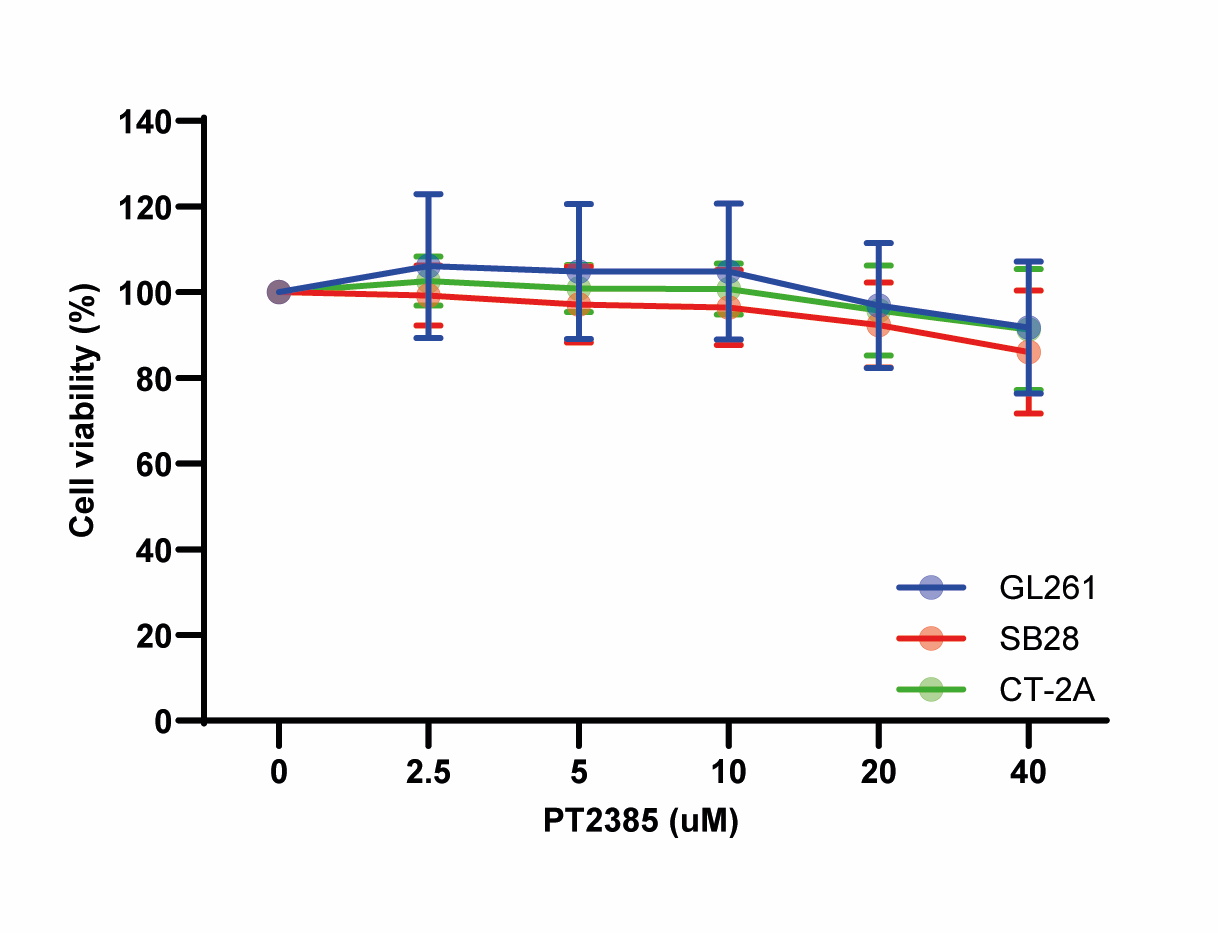


**Supplementary Figure 1. HIF-2α inhibition does not affect the viability of cells from 3 mouse glioma cell lines.** Cell viability was measured by CellTiter/Glo® assay, based on ATP content by relative light unit (RLU). Mouse glioma cell lines GL261, SB28, and CT-2A were incubated with PT2385 for 24 hours from 0 to 40 μM in normoxic oxygen tensions (21% 0_2_). Graphs represent the mean of percentage normalized to vehicle control (0 μM PT2385) ± SD, n=5.


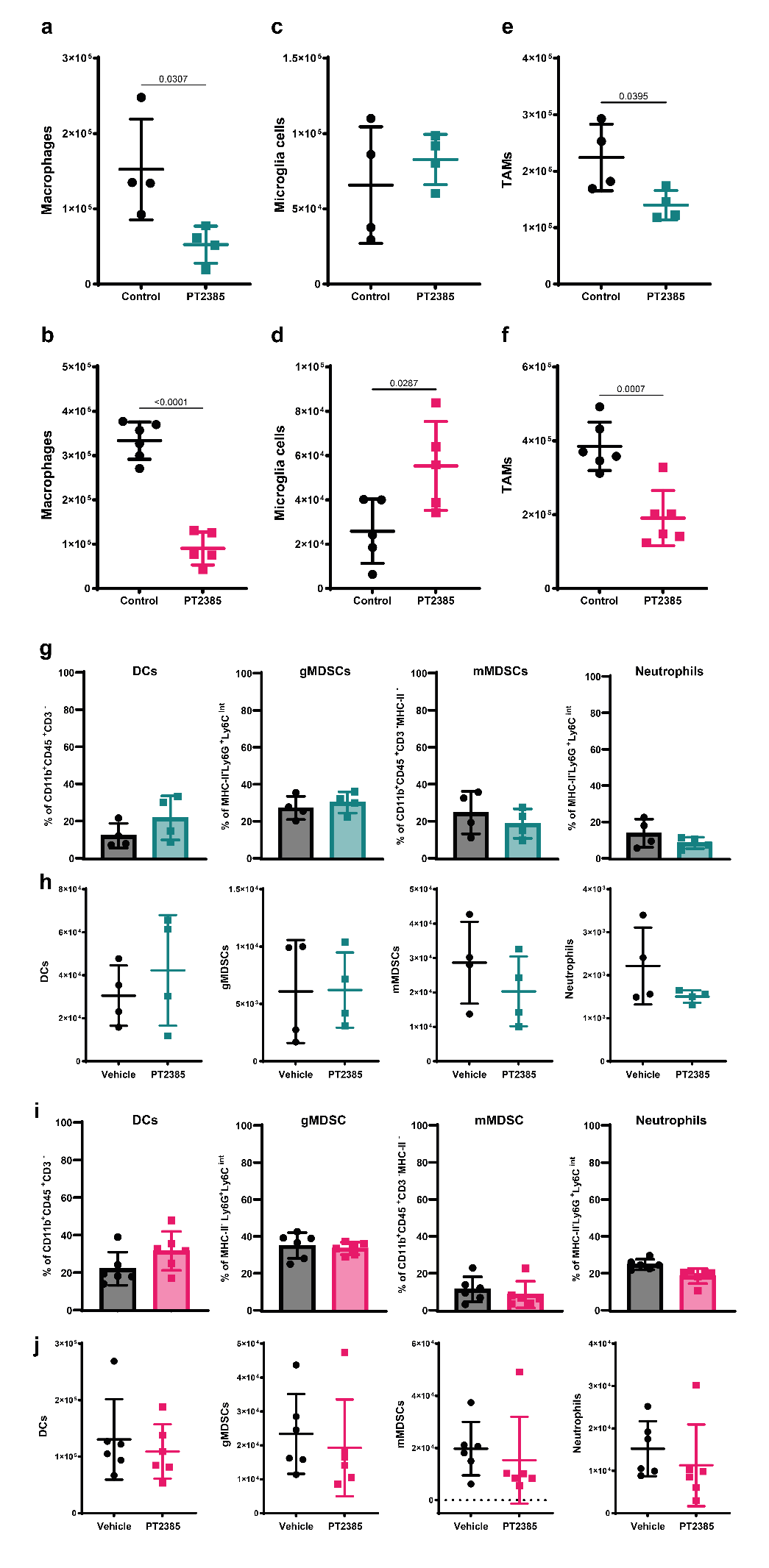


**Supplementary Figure 2. HIF-2α inhibition significantly decreases macrophage and increases microglial cell counts within the glioma microenvironment.** Flow cytometry quantification of numbers of macrophages and microglia. Live cells were gated on CD45^+^CD11b^+^CD3^-^ cells. Graphs show mean ± SD, with sample sizes of n=4 for midterm (green) and n=6 for endterm (pink); p-values were calculated using an unpaired t-test. Estimated total cell number per brain was calculated by normalizing the counts of live cells and total cell counts. At midterm (green) and endterm (pink): **(a, b)** macrophages, **(c, d)** microglia, **(e, f)** microglia and macrophages. (**g**) proportions at midterm of DCs, gMDSCs, mMDSCs, and neutrophils. **(h)** estimated cell number at midterm of DCs, gMDSCs, mMDSCs, and neutrophils. (**g**) proportions at endterm of DCs, gMDSCs, mMDSCs, and neutrophils. **(h)** estimated cell number at endterm of DCs, gMDSCs, mMDSCs, and neutrophils.


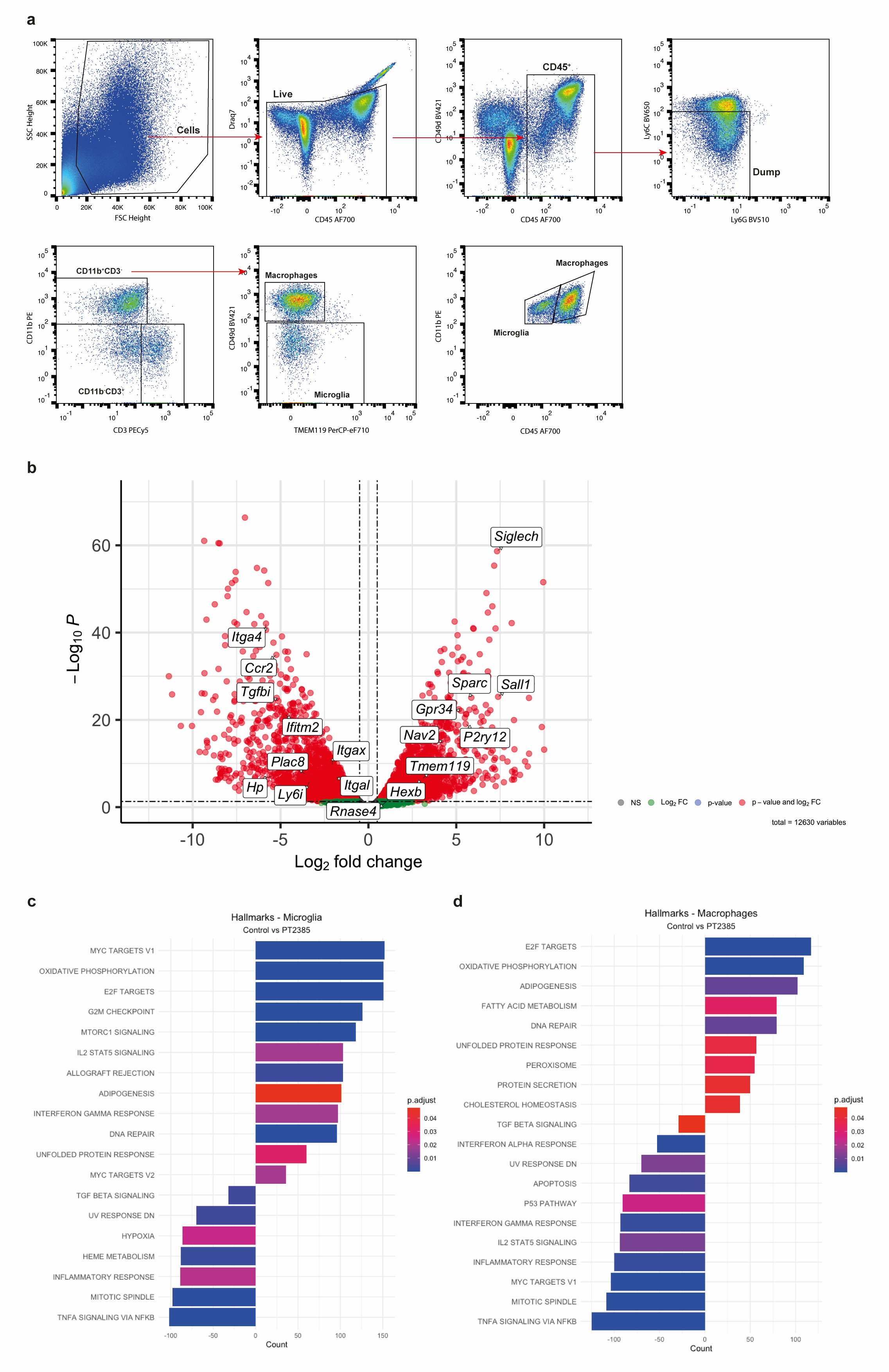


**Supplementary Figure 3. Bulk RNA-seq of sorted microglia and macrophages. (a)** Representative plots of flow cytometry gating to sort microglia and macrophages from GL261-bearing brains at day 21 **(b)** Vulcano plots of differential gene expression analysis in sorted microglia versus macrophages in samples treated with vehicle (Red: Padj < 0.05, LFC > 0.5 or LFC < -0.5, green: LFC > 0.5 or LFC < -0.5 and grey: not significant). **(d-e)** Gene set enrichment analysis (GSEA) of hallmark gene sets. The y-axis lists the upregulated and downregulated enriched hallmarks, the count of genes per hallmark in the X-axis, and the gradient color indicates the p.adj value.


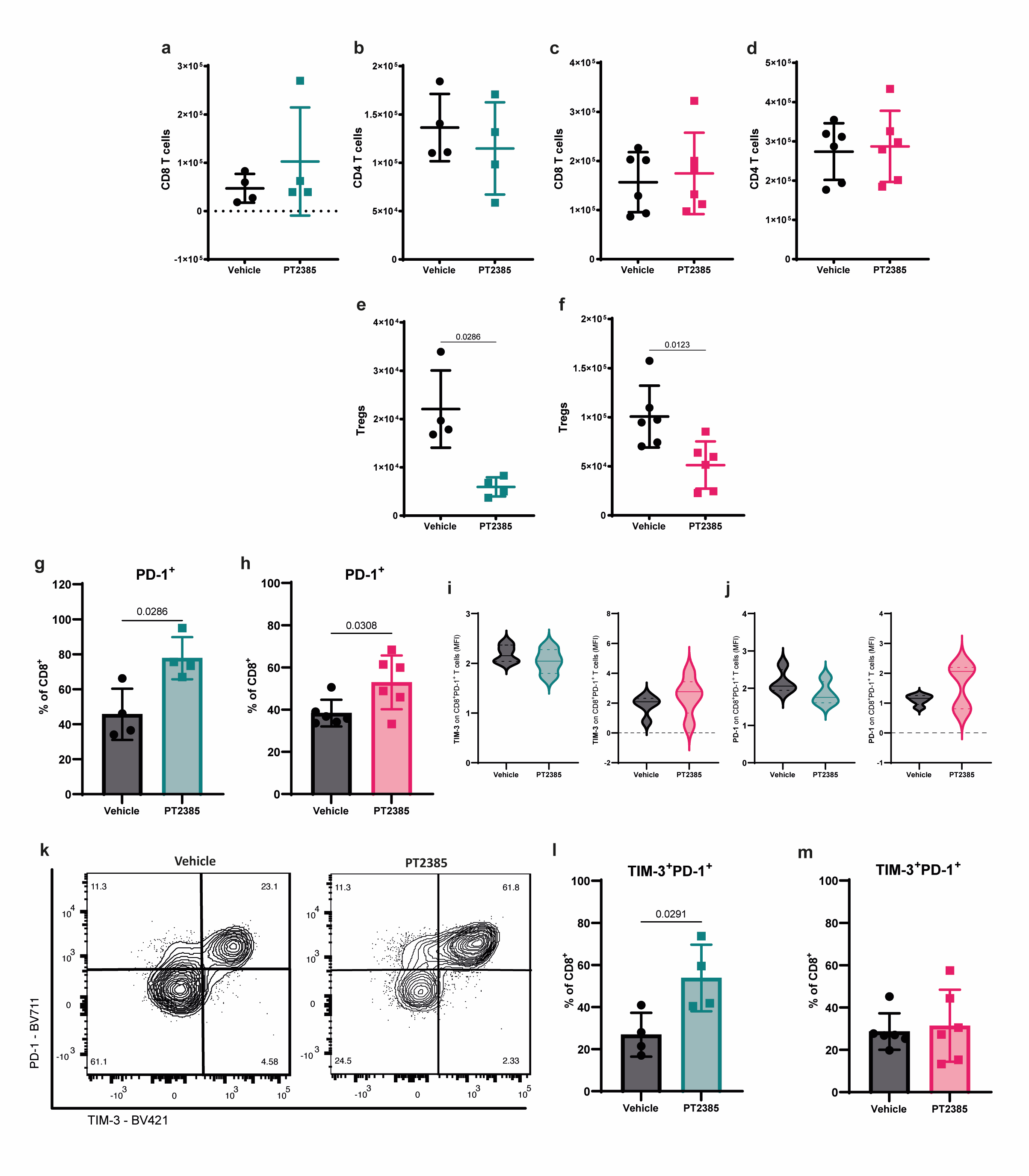


**Supplementary Figure 4. HIF-2α inhibition significantly decreases Treg counts.** Quantification of the T cell populations of GL261-bearing brain mice treated with vehicle or PT2385 at midterm (green) and endterm (pink). Live cells were gated on CD45^+^CD11b^-^CD3^+^ cells. Estimated total cell number per brain was calculated by normalizing the counts of live cells and total cell counts. **(a)** CD8^+^ T cell counts at midterm, **(b)** CD4^+^ T cell counts at midterm, **(c)** CD8^+^ T cell counts at endterm, **(d)** CD4^+^ T cell counts at endterm, **(e)** Treg counts at midterm, and **(f)** Treg counts at endterm. Proposition of PD-1^+^ cells among CD8^+^ T cells at **(g)** midterm and **(h)** endterm. **(i)** mean fluorescence intensity (MFI) of TIM-3 expression by CD8^+^/PD-1^+^ T cells. **(j)** MFI of PD-1 expression by CD8^+^PD-1^+^ T cells. **(k)** Representative plots of PD-1 and TIM-3 expression gated on CD8^+^ T cells at midterm in vehicle- and PT2385-treated mice. **(l-m)** Proportions of PD-1^+^/TIM-3^+^ among CD8^+^ T cells at midterm **(l)** or endterm **(m).** Graphs show mean ± SD, n=4 (midterm), n=6 (endterm), and p-value calculated using unpaired t-test.


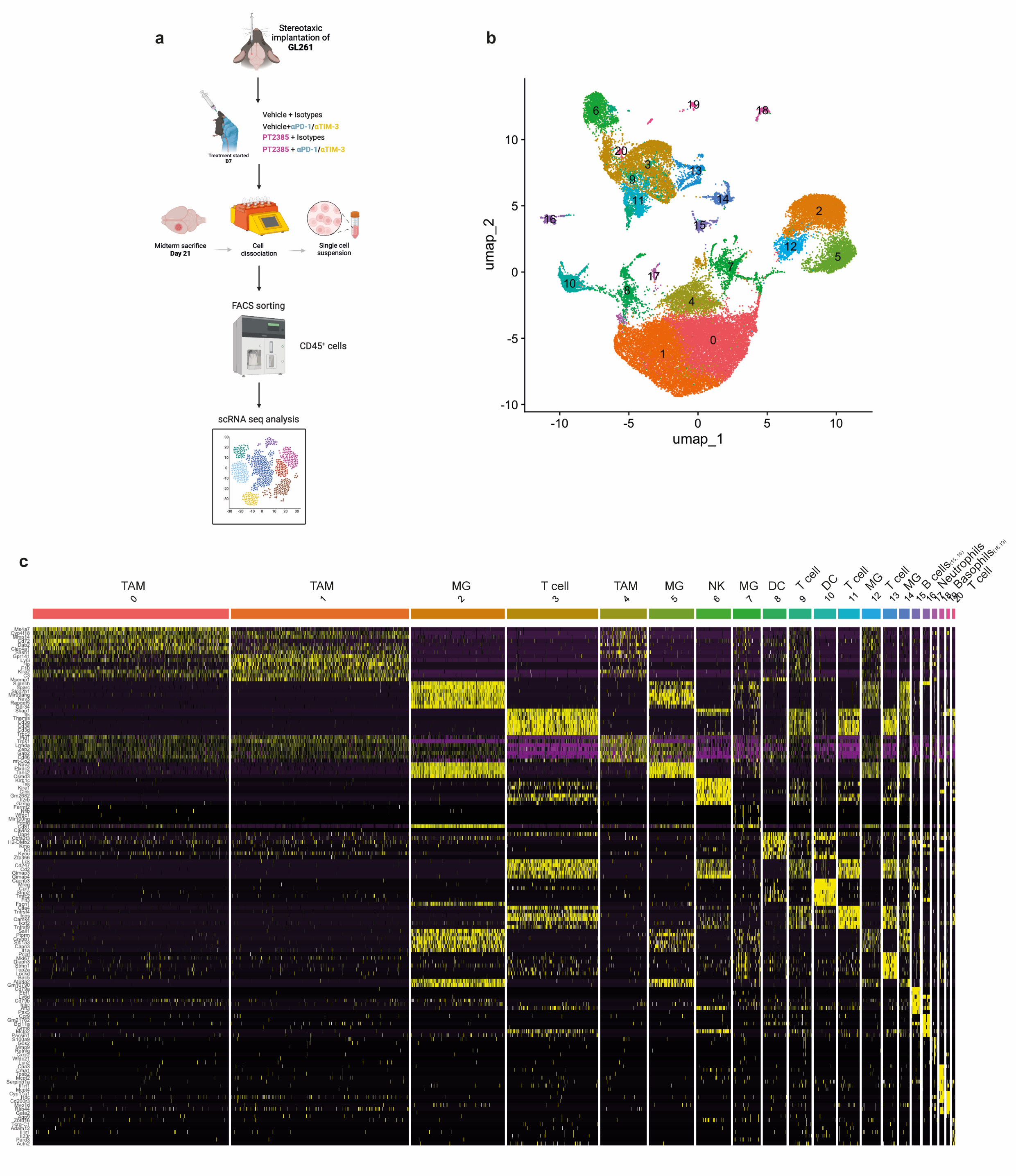


**Supplementary Figure 5. scRNAseq of tumor-infiltrating leukocytes in GL261-bearing mice.** **(a)** Schematic representation of mice treatment, sample processing, and 10X Genomics analysis. **(b)** UMAP for dimensionality reduction representing the cell cluster of the integrated data set of all samples obtained during unsupervised clustering in Seurat. **(c)** Heatmap representing expression level for the top 10 enriched genes in each cluster.


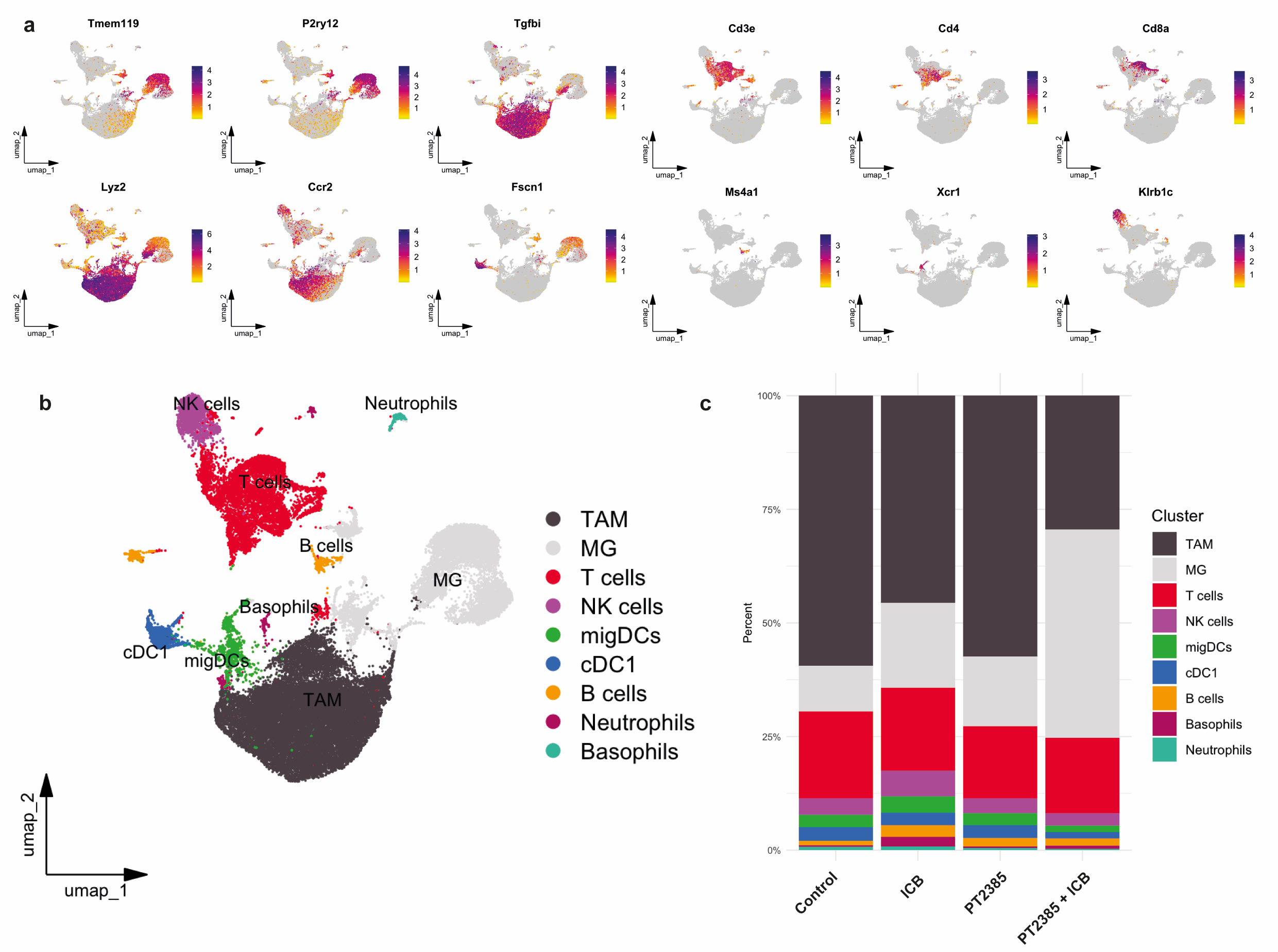


**Supplementary Figure 6.** **Leukocytes clusters in GL261-bearing mice across all conditions.** **(a)** Feature plots showing canonical gene markers expression for each cell cluster in the UMAP plot. **(b)** UMAP plot of all identified clusters through manual annotation. **(c)** Proportions of cell clusters in each group (from left to right, isotype control, dual ICB, PT2385, and PT2385 plus dual ICB).


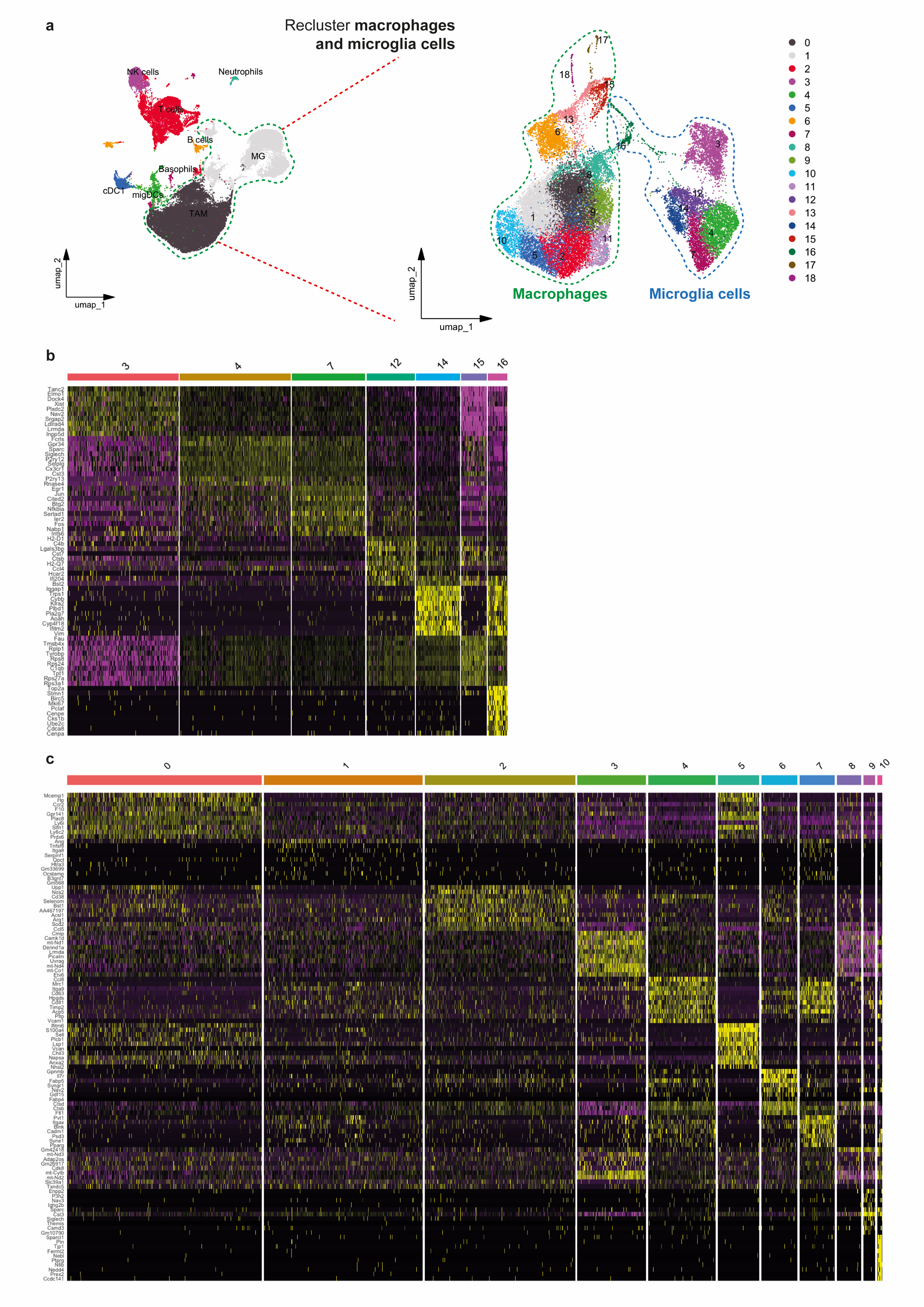


**Supplementary Figure 7. Subset and recluster of microglia and macrophage clusters.** **(a)** Schematic representation of the subset cluster identified as TAM and MG to perform de novo unsupervised clusterization. **(b)** Heatmap representing expression level for the top 10 enriched genes in each microglia cluster. **(c)** Heatmap representing expression level for the top 10 enriched genes in each microglia cluster.


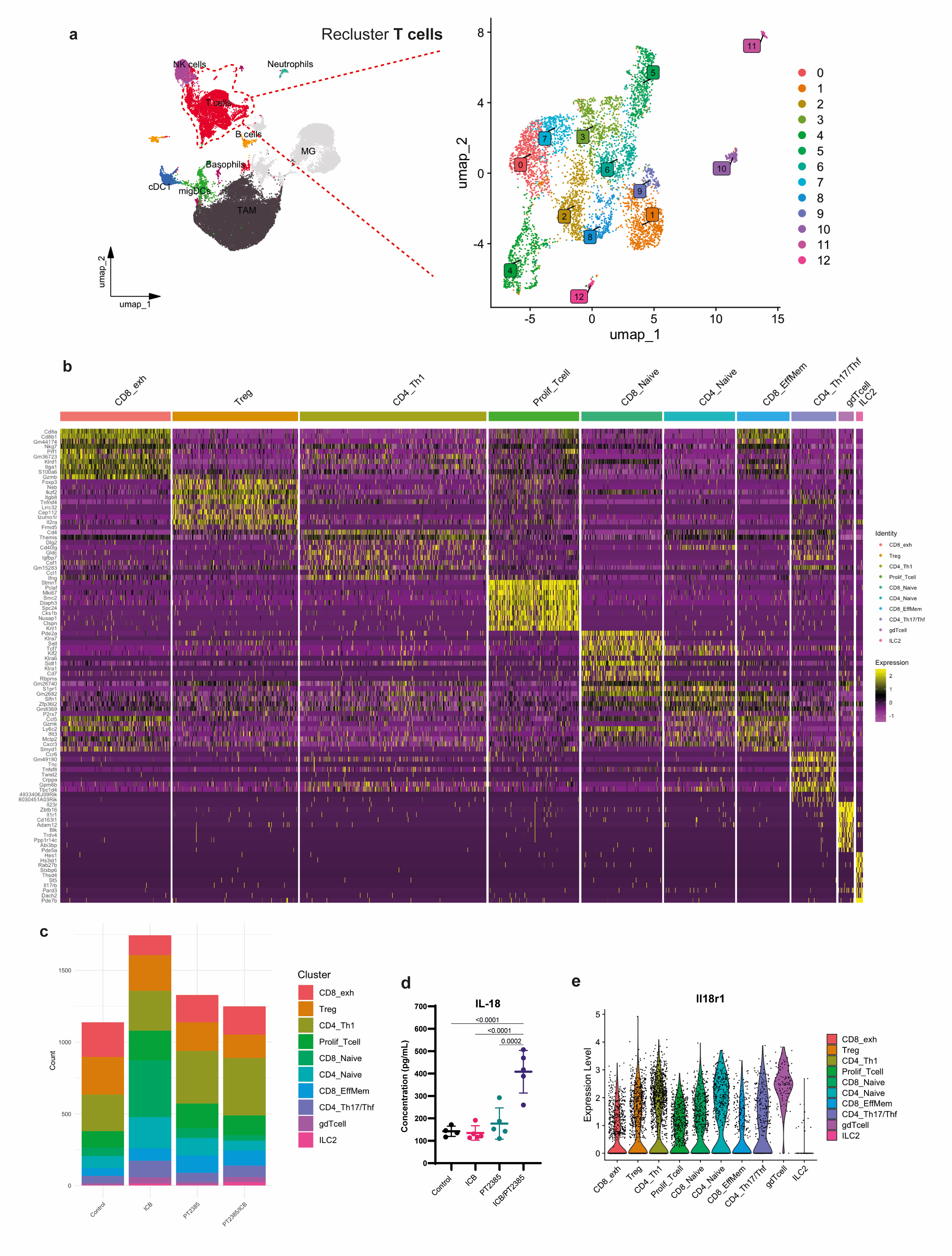


**Supplementary Figure 8. Subset and recluster of T cell clusters.** **(a)** Schematic representation of the subset cluster identified as T cell to perform de novo unsupervised clusterization. **(b)** Heatmap representing expression level for the top 10 enriched genes in each T cell cluster following manual annotation. **(c)** Total count of all T cell clusters following manual annotation. **(d)** IL-18 quantification in cells from dissociated GL261-bearing brains at midterm. The assay was performed using a multiple-analyte flow assay kit (Legendplex). Graphs show mean ± SD, n=5 per group, and statistics by one-way ANOVA test. **(e)** *Il18r1* mRNA expression in the identified T cell cluster through manual annotation.


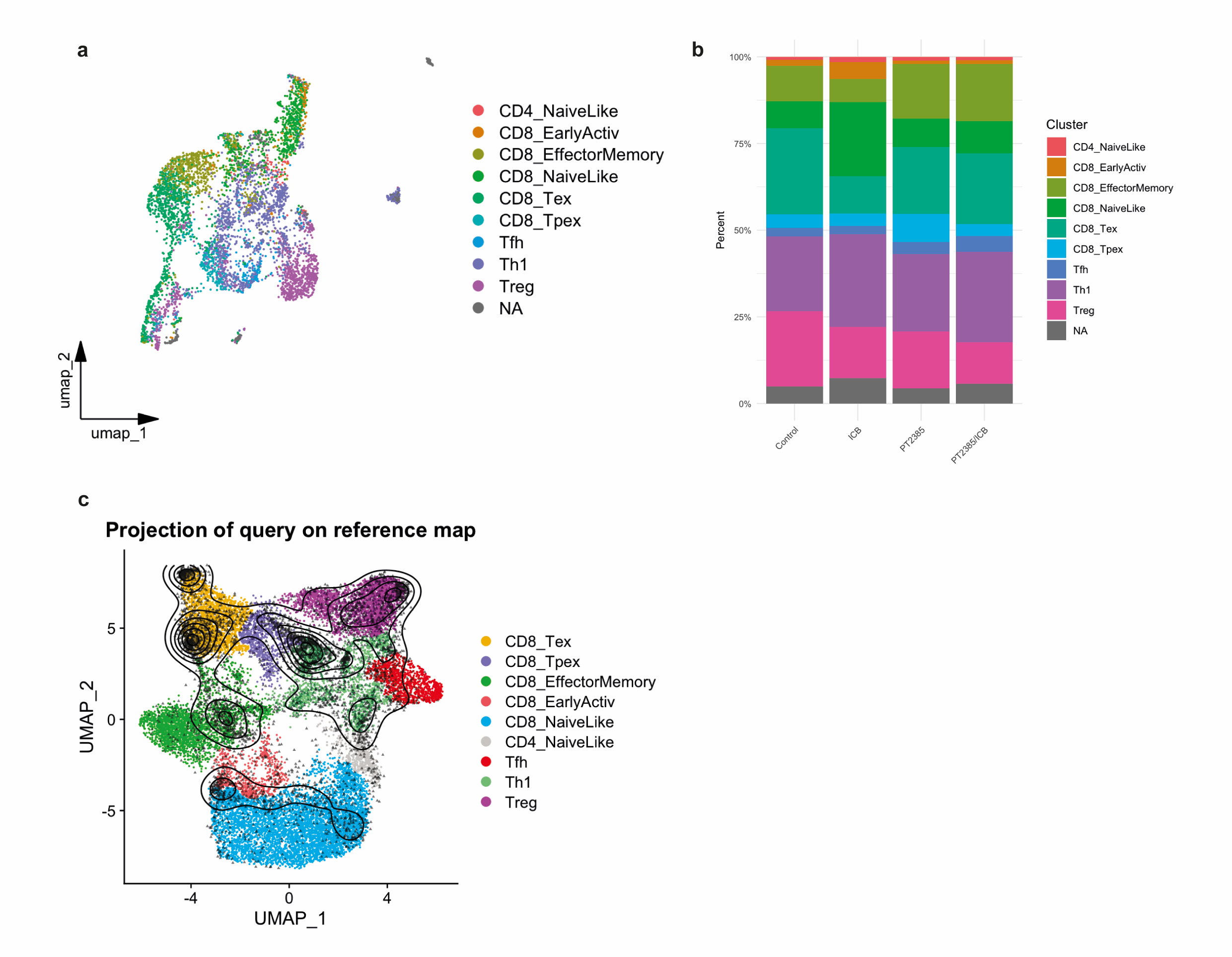


**Supplementary Figure 9. T cell subpopulation clusterized through atlas-based annotations.** **(a)** UMAP projection of original space data labeled with a previously annotated system of coordinates using a mouse single-cell atlas of TILs. **(b)** Proportions of all T cells cluster following atlas-based annotation method. **(c)** UMAP projection of projected scRNA-seq data into the reference map of the mouse single-cell atlas of TILs


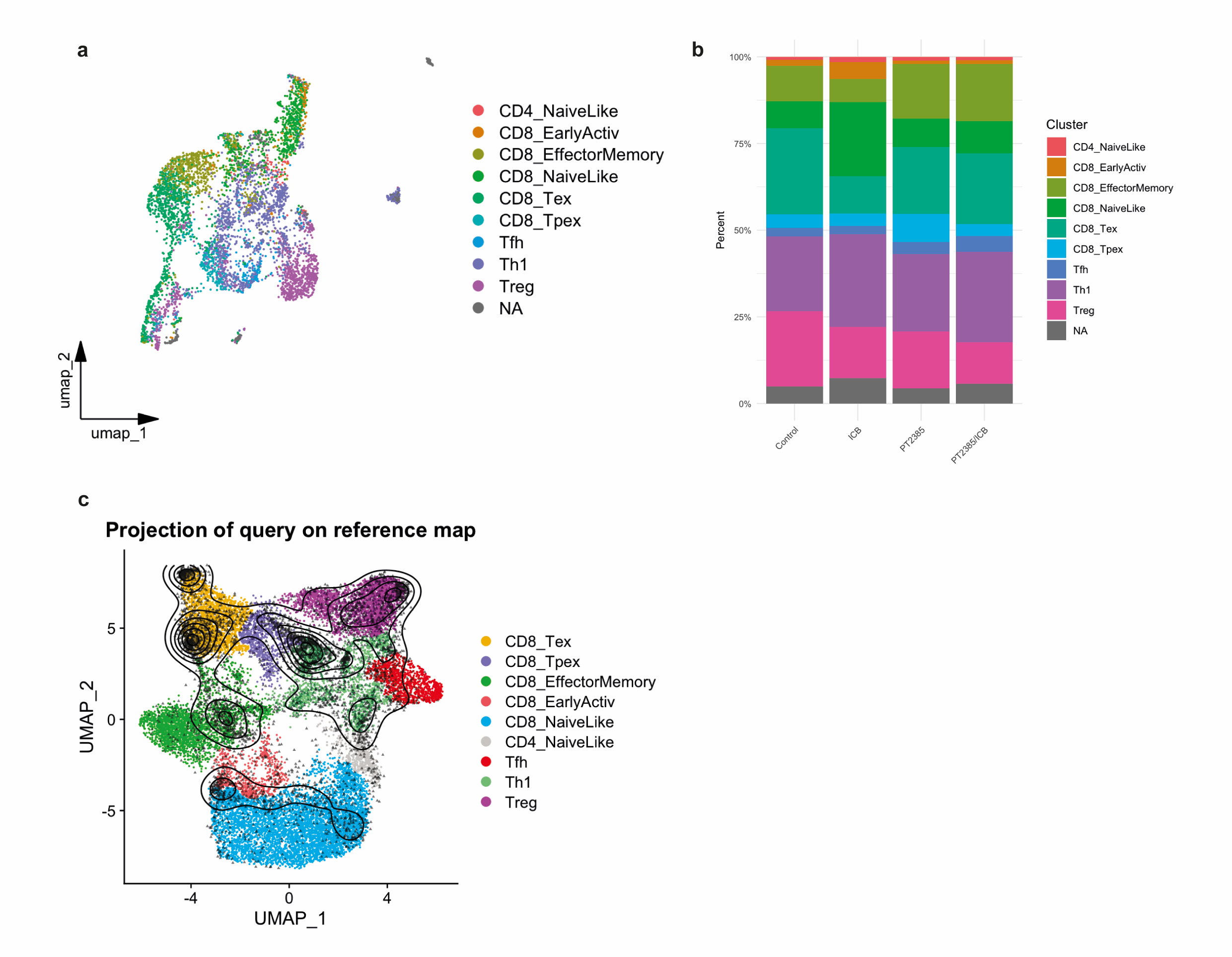


**Supplementary Figure 10. HIF-2α inhibition combined with ICB trends to increase CD8^+^ T cell numbers and decrease numbers of Tregs.** Flow cytometry quantification of lymphocyte populations of GL261-bearing mice at midterm. Live cells were gated on CD45^+^CD11b^-^CD3^+^ cells. Absolute cell numbers were calculated by normalizing the counts of beads and total cell counts per tumor-bearing hemisphere. **(a)** CD4^+^ T cell numbers at midterm, **(b)** CD8^+^ T cell numbers at endterm, and **(c)** Treg (FOXP3^+^CD25^+^) numbers at midterm. **(f)** Proportions Granzyme B^+^ cells 3^-^ of CD4^+^ T cells at midterm **(g)** Representative gating plots of Granzyme B and FOXP3 proportions in all groups. **(f)** Proportions progenitor-exhausted CD8^+^ T cells defined as SLAMF6^+^TIM-3^-^ of CD8^+^PD-1^+^ T cells at midterm **(g)** Representative gating plots of SLAMF6 and TIM-3 proportions in all groups. Graphs show mean ± SD, n=5 per group, and statistics by one-way ANOVA test.


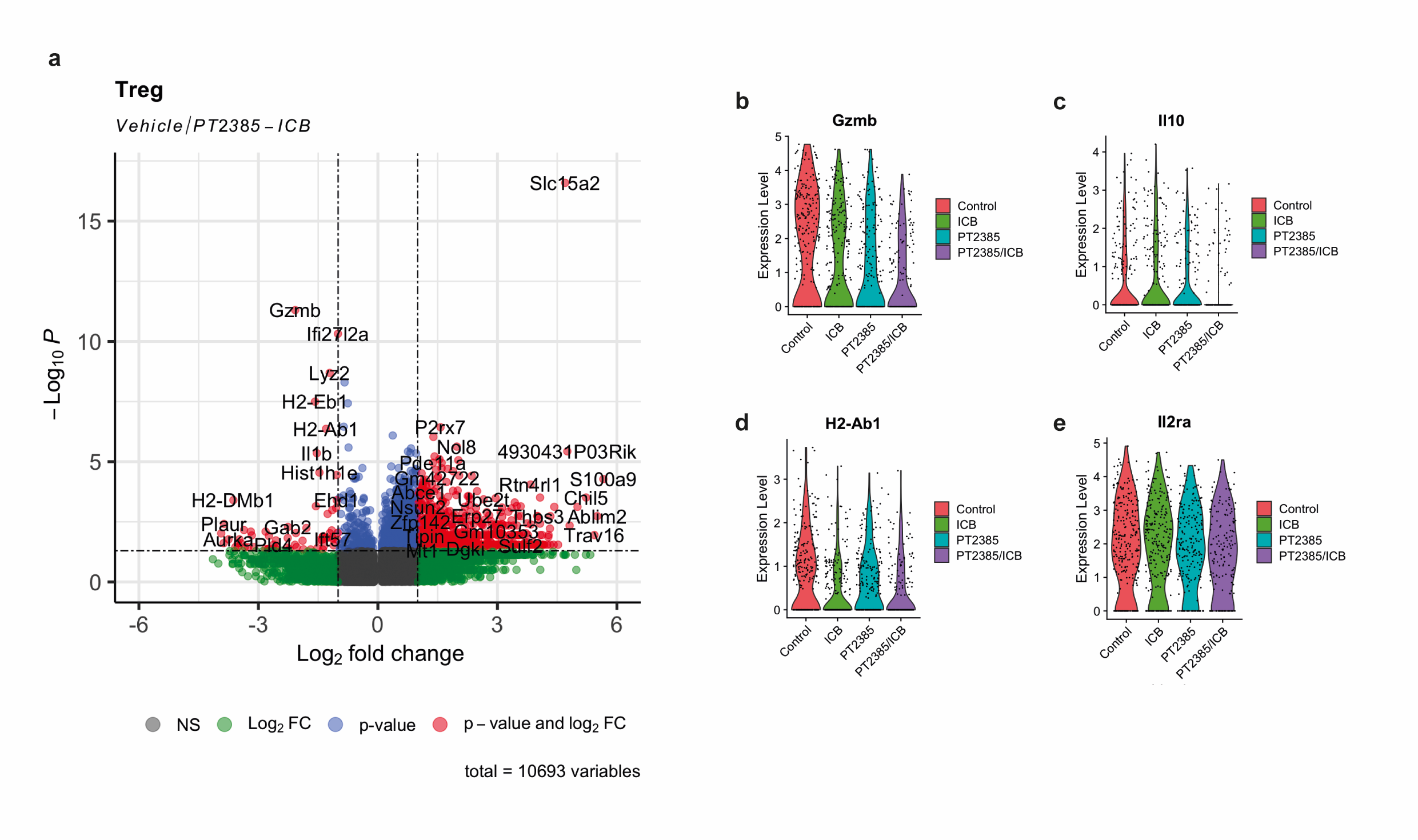


**Supplementary Figure 11. HIF-2α inhibition combined with ICB downregulates immunosuppressive molecules in the Treg cluster (a)** Vulcano plots of differential expression gene (DEG) analysis in the Treg cluster from vehicle/isotypes-treated mice versus PT2385/ICB-treated mice (Red: Padj < 0.05, LFC > 0.5 or LFC < -0.5, green: LFC > 0.5 or LFC < -0.5 and grey: not significant). **(b)** *Il18r1*, **(c)** *Il10*, **(d)** *H2-Ab1*, and **(e)** *Il2ra* mRNA expression levels in the Treg cell cluster in each combinatory treatment group.


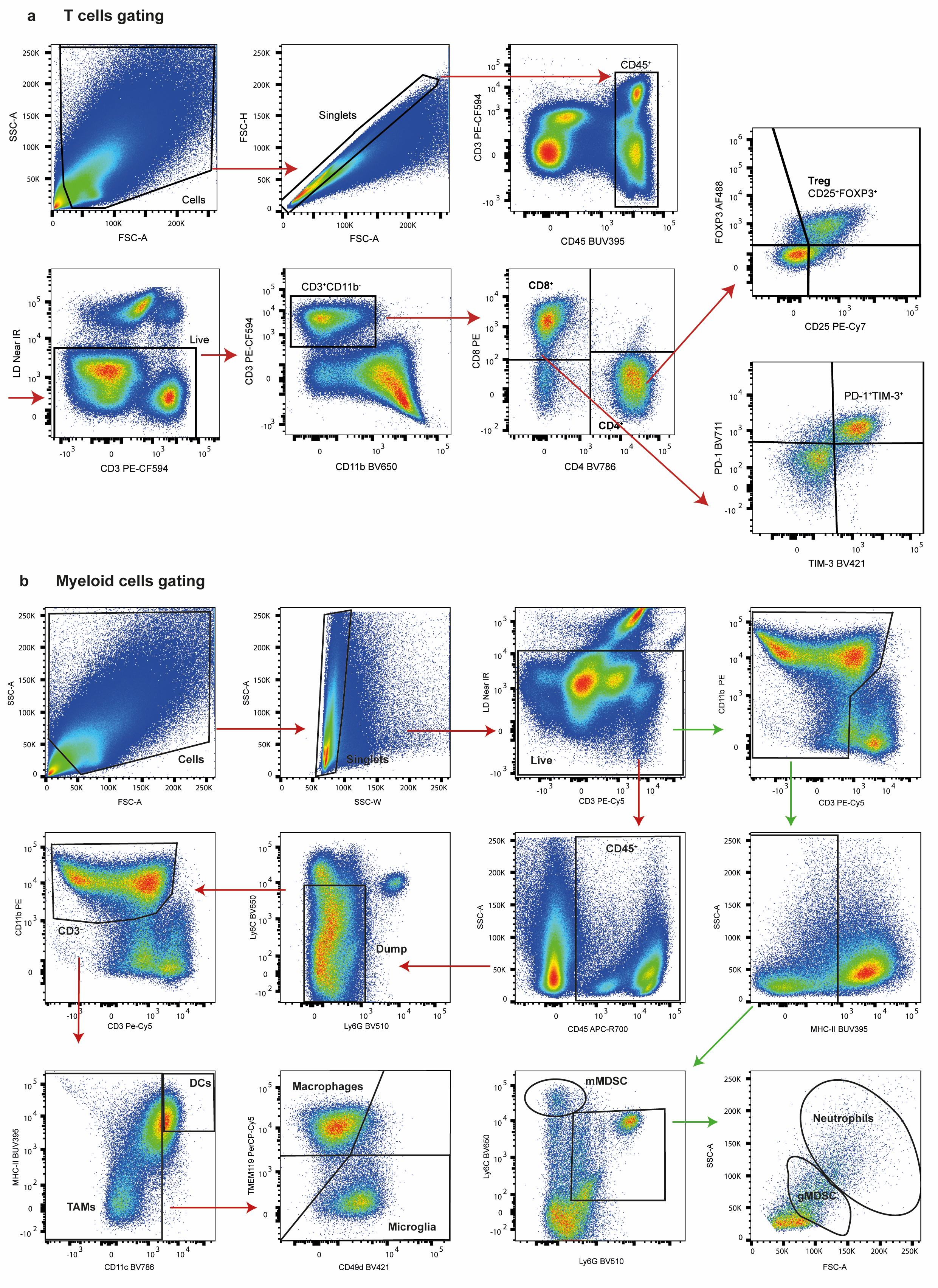


**Supplementary Figure 12. Flow cytometry gating of brain-infiltrating leukocytes (a)** Representative plots of flow cytometric gating of T cells of brain-infiltrating leukocytes in GL261-bearing mice. **(b)** Representative plots of flow cytometric gating of myeloid of brain-infiltrating leukocytes in GL261-bearing
